# Association of Race/Ethnicity and Social Disadvantage With Autism Prevalence in 7 Million School Children in England

**DOI:** 10.1001/jamapediatrics.2021.0054

**Published:** 2021-03-29

**Authors:** Andres Roman-Urrestarazu, Robin van Kessel, Carrie Allison, Fiona E. Matthews, Carol Brayne, Simon Baron-Cohen

**Affiliations:** 1 Autism Research Centre, Department of Psychiatry, University of Cambridge, Cambridge, United Kingdom; 2 Department of International Health, School CAPHRI (School for Public Health and Primary Care), Faculty of Health, Medicine and Life Sciences, Maastricht University, Maastricht, the Netherlands; 3 Cambridge Public Health, University of Cambridge, Cambridge, United Kingdom; 4 Population Health Sciences Institute, Newcastle University, Newcastle, United Kingdom

## Abstract

**Importance:**

The global prevalence of autism spectrum disorder (ASD) has been reported to be between 1% and 2% of the population, with little research in Black, Asian, and other racial/ethnic minority groups. Accurate estimates of ASD prevalence are vital to planning diagnostic, educational, health, and social care services and may detect possible access barriers to diagnostic pathways and services and inequalities based on social determinants of health.

**Objective:**

To evaluate whether socioeconomic disadvantage is associated with ASD prevalence and the likelihood of accessing ASD services in racial/ethnic minority and disadvantaged groups in England.

**Design, Setting, and Participants:**

This case-control prevalence cohort study used the Spring School Census 2017 from the Pupil Level Annual Schools Census of the National Pupil Database, which is a total population sample that includes all English children, adolescents, and young adults aged 2 to 21 years in state-funded education. Data were collected on January 17, 2017, and analyzed from August 2, 2018, to January 28, 2020.

**Exposures:**

Age and sex were treated as a priori confounders while assessing correlates of ASD status according to (1) race/ethnicity, (2) social disadvantage, (3) first language spoken, (4) Education, Health and Care Plan or ASD Special Educational Needs and Disability support status, and (5) mediation analysis to assess how social disadvantage and language might affect ASD status.

**Main Outcomes and Measures:**

Sex- and age-standardized ASD prevalence by race/ethnicity and 326 English local authority districts in pupils aged 5 to 19 years.

**Results:**

The final population sample consisted of 7 047 238 pupils (50.99% male; mean [SD] age, 10.18 [3.47] years) and included 119 821 pupils with ASD, of whom 21 660 also had learning difficulties (18.08%). The standardized prevalence of ASD was 1.76% (95% CI, 1.75%-1.77%), with male pupils showing a prevalence of 2.81% (95% CI, 2.79%-2.83%) and female pupils a prevalence of 0.65% (95% CI, 0.64%-0.66%), for a male-to-female ratio (MFR) of 4.32:1. Standardized prevalence was highest in Black pupils (2.11% [95% CI, 2.06%-2.16%]; MFR, 4.68:1) and lowest in Roma/Irish Travelers (0.85% [95% CI, 0.67%-1.03%]; MFR, 2.84:1). Pupils with ASD were more likely to face social disadvantage (adjusted prevalence ratio, 1.61; 95% CI, 1.59-1.63) and to speak English as an additional language (adjusted prevalence ratio, 0.64; 95% CI, 0.63-0.65). The effect of race/ethnicity on ASD status was mediated mostly through social disadvantage, with Black pupils having the largest effect (standardized mediation coefficient, 0.018; *P* < .001) and 12.41% of indirect effects through this way.

**Conclusions and Relevance:**

These findings suggest that significant differences in ASD prevalence exist across racial/ethnic groups and geographic areas and local authority districts, indicating possible differential phenotypic prevalence or differences in detection or referral for racial/ethnic minority groups.

## Key Points

**Question:**

What is the prevalence of autism spectrum disorder (ASD) in the total English state school population, and what are the social determinants associated with ASD status?

**Findings:**

In this ASD prevalence cohort study of 7 047 238 pupils, national English prevalence was 1.76%, with marked differences according to racial/ethnic group. The highest prevalence was found in Black pupils (2.11%) and the lowest in Roma/Irish Travelers (0.85%), with important variability across geographic areas.

**Meaning:**

These results show differences in ASD prevalence estimates across racial/ethnic minority groups in England, which could be attributable to diagnostic biases, possible differences in detection and referral, or differential phenotypic prevalence for racial/ethnic minority groups.

## Teaser

This national cohort study evaluates whether socioeconomic disadvantage is associated with autism spectrum disorder prevalence and the likelihood of accessing autism services in racial/ethnic minority groups and disadvantaged groups among school pupils in England.

## Introduction

Autism spectrum disorder (ASD) is a cluster of neurodevelopmental conditions characterized by persistent difficulties in social communication and interaction, and restricted, repetitive patterns of behavior, interests, or activities across multiple contexts.^1,2,3^ The global prevalence of ASD is between 1% and 2% of the population, with a reported male-to-female ratio (MFR) of 3:1 or 2:1.^1,2,4,5^ Discussions about a possible increase in ASD prevalence have pointed to its shifting diagnostic features,^6,7^ with little research in Black, Asian, and racial/ethnic minority groups.^8,9,10^ Accurate estimates of ASD prevalence are vital to planning diagnostic, educational, health, and social care services and identifying possible access barriers to diagnostic pathways and services and inequalities based on social determinants of health, such as socioeconomic disadvantage, race/ethnicity, and or sex.

In this ASD prevalence study, which to our knowledge is the largest to date, we address the aforementioned gaps using the Spring School Census 2017 from the National Pupil Database (NPD) in England, which is an administrative data source. We estimated ASD prevalence in English schools to explore the association between ASD and sociodemographic and socioeconomic factors or characteristics.^11,12,13^ We stratified the prevalence estimates by sex; race/ethnicity; Special Educational Needs and Disability (SEND) status, which is an assessment determining whether a pupil requires special educational provision^14^; and Local Authority Districts (henceforth districts) and then calculated MFRs nationally and by locality. We then sought to identify possible access barriers to SEND services in ASD by stratifying our outcomes by race/ethnicity, the Free School Meals (FSM) program (used as a proxy for socioeconomic disadvantage), and first language spoken (as a measure of social inclusion) in pupils aged 5 to 19 years. We hypothesized that social determinants of health and experiences of multiple socioeconomic disadvantage and identity- or culture-based forms of alienation would influence the likelihood that people in disadvantaged and racial/ethnic minority groups will access ASD services.

## Methods

### The NPD and Data Access

The NPD is a total school population registry collected in England by the Department for Education that maintains counts of all pupils aged 2 to 21 years under State education provision. One of its components is the Pupil Level Annual Schools Census, which is conducted every term. For the purpose of this project, we used the Spring School Census 2017, which was collected January 17, 2017. Data access was granted by the Department for Education in March 2018 and approved by the Ethics Committee from the Department of Psychology, University of Cambridge, Cambridge, United Kingdom. Informed consent was not needed for this publicly available data source. This study followed the Strengthening the Reporting of Observational Studies in Epidemiology (STROBE) reporting guideline. Details of the NPD are provided in the eMethods in the Supplement.

### Special Educational Needs and Disabilities in England

The care that children and young people receive in English schools varies according to SEND status. Two levels of provision are in place in England: SEND support, which is a school-specific learning program given to a pupil; and Education, Health and Care Plans (EHCPs; previously known as statements), which were introduced as part of the Children and Families Act 2014.^14^ Currently, the School Census includes 14 support/EHCP categories: (1) specific learning difficulty; (2) moderate learning difficulty; (3) severe learning difficulty; (4) profound and multiple learning difficulties; (5) speech, language, and communication needs; (6) hearing impairment; (7) visual impairment; (8) multisensory impairment; (9) physical disability; (10) ASD; (11) other difficulty/disability; (12) social, emotional, and mental health; (13) SEND support but no specialist assessment; and (14) unclassified. The EHCP needs assessments are usually performed by local authorities with assistance from the National Health Service, Child and Adolescent Mental Health Services, which manages the ASD diagnostic pathway across England. The NPD SEND registry includes as many as 2 SEND categories per pupil.

### Operationalizing ASD Status From Administrative Educational Data

To operationalize ASD by using SEND registry data contained in the Spring School Census, we coded ASD if it was a primary or secondary SEND and included (1) an EHCP, which contains F84.0 to F84.9 ASD diagnosis codes from the *International Statistical Classification of Diseases and Related Health Problems, Tenth Revision,*^15^ and (2) school support given to pupils by educators. Autism spectrum disorder SEND support therefore includes pupils who require access to additional ASD-specific support beyond the school’s differentiated curriculum, which also encompasses pupils awaiting their EHCP.^15^ We calculated the prevalence for these 2 different SEND provision categories separately and then created a composite third variable that was coded by merging pupils with an ASD EHCP and those pupils receiving SEND support to calculate the standardized prevalence of ASD status in the English educational system. This variable is the main outcome for our prevalence estimates and, for the purpose of this study, defines pupils as having documented ASD status in English schools. We assume that this composite variable of ASD status is the most representative estimate of formally recognized ASD prevalence in the English educational system.

### Sociodemographic Variables and Regional Analysis Units

Individuals were categorized into 3 age groups (5-9, 10-14, and 15-19 years). Sex was binarily coded. The NPD has 8 self-reported major categories of race/ethnicity, including (1) any other racial/ethnic group, (2) Asian, (3) Black, (4) Chinese, (5) mixed, (6) unclassified, (7) White, and (8) Roma/Irish Traveler, all of which are based on the English and Welsh census categories. First language spoken was coded as English or other. The NPD defines this as the language to which the child was exposed during early development and continues to use in the home or in the community regardless of subsequent English proficiency. We used dichotomized lifetime claimed eligibility to the FSM program as a proxy of socioeconomic disadvantage.^16^ England has 326 districts, which comprise a level subnational division of England used for the purposes of local government. We included all 326 districts for our analysis. We also created a novel outcome ratio that we have termed the *standardized statement–to-support ratio* (SSR). The SSR is used to describe the proportion of EHCPs vs support as a standardized rank to assess how districts perform in processing EHCPs. The higher the SSR, the higher the proportion of ASD EHCPs compared with ASD SEND support. Further details are included in the eMethods in the Supplement.

### Statistical Analyses, Multiple Imputation for Missing Data, and Sensitivity Analyses

Data were analyzed from August 2, 2018, to January 28, 2020. Raw prevalence estimates for ASD were directly standardized and stratified by age group, sex, and districts using the English 2011 census projections for 2017 as a standard population to calculate national prevalence by race/ethnicity and sex and prevalence across 326 districts by sex, using the World Health Organization standard method.^17,18,19^ Adjusted relative risk (referred to henceforth as adjusted prevalence ratio [aPR]) estimates were obtained using a Poisson regression with robust error variance to evaluate access to services using the following outcome variables: (1) pupils with ASD, (2) pupils with ASD who had SEND support, (3) pupils with ASD who had an EHCP, and (4) pupils with ASD who had a second SEND. In each outcome model, we used the same independent variables of age, sex, race/ethnicity, first language spoken, and socioeconomic disadvantage (through the FSM program) and included them in the same adjusted model comparing all levels against each other and reporting for missing data. After this initial approach and identification of missingness, we optimized model fit while accounting for missing data by using multiple imputation by chained equations in our Poisson regression with robust error variance, which uses a separate conditional distribution for each imputed variable.^20^ To avoid bias in the imputation model, we included all variables listed in the main analyses.^20^ To control for multiple comparisons, we used a significance level of 2-sided *P* < .001 for all reported outcomes. We then conducted a 1-way sensitivity analysis comparing the multiple imputation by chained equations ASD model with the Poisson regression with a robust error variance model and a log-binomial regression. The comparisons were performed against a full data model and a complete analysis model for the variables with missing data, checking for model fit and effect on statistical significance.^20^

For the mediation analysis, which was used to evaluate the role that race/ethnicity and socioeconomic disadvantage plays in ASD status, we included a dichotomized ASD status as the outcome, a dichotomized claimed eligibility to the FSM program and a dichotomized variable of language spoken as mediators, and race/ethnicity as our independent variable, coded as 7 dummy variables using White pupils as our comparator group.^21^ We applied a planned regression approach to evaluate associations among the dependent, independent, and mediating variables.^21^ We included age group and sex as covariates of interest. We then used a weighted least-square means and variance-adjusted model design used for categorical data. In this analysis, SEs for the standardized path coefficients are not computed (eFigure 6 in the Supplement). Consequently, we only report the standardized estimates and the respective bootstrapped 95% CIs with *P* values.

## Results

### Descriptive Statistics

Our final sample included 7 047 238 pupils, of whom 50.99% were male and 49.01% were female (mean [SD] age, 10.18 [3.47] years). A total of 119 821 pupils with ASD were identified in our sample, of whom 21 660 (18.08%) were also classified as having a learning difficulty. Among pupils with ASD without a learning difficulty, 65.41% were recorded with a second SEND. With regard to socioeconomic disadvantage, 25.80% of all English school pupils (n = 1 818 195) had ever claimed eligibility for the FSM program. When comparing socioeconomic disadvantages in the school population, we found that pupils with ASD (35.23%), pupils with ASD and a learning difficulty (37.93%), and pupils with other types of SEND (44.61%) all had higher proportions of socioeconomic disadvantage than pupils with no SEND (24.47%). A total of 18.51% of pupils spoke a language other than English first, with other ethnic group (80.47%), Asian (73.77%), Black (43.68%), Chinese (75.89%), and Roma/Irish Traveler (44.65%) having the largest proportion of pupils in this category. All descriptive statistics are presented in eTable 1 in the Supplement.

### National Prevalence by Race/Ethnicity, Sex, EHCP, and Support Status

**Figure 1.**
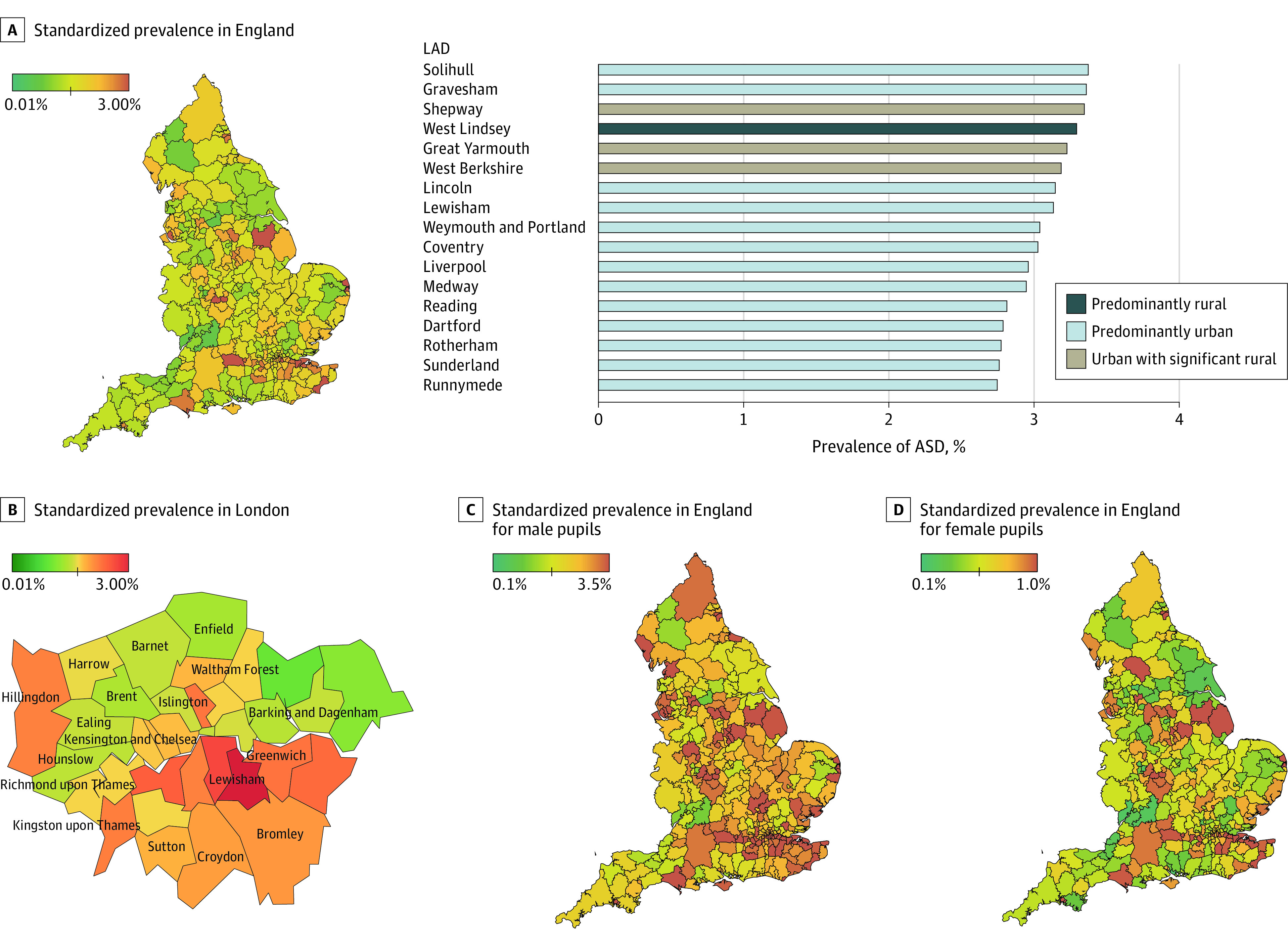
Heat Maps of Autism Spectrum Disorder (ASD) Prevalence by Local Authority District (LAD) in England Data are stratified by district, sex, and age.

**Figure 2.**
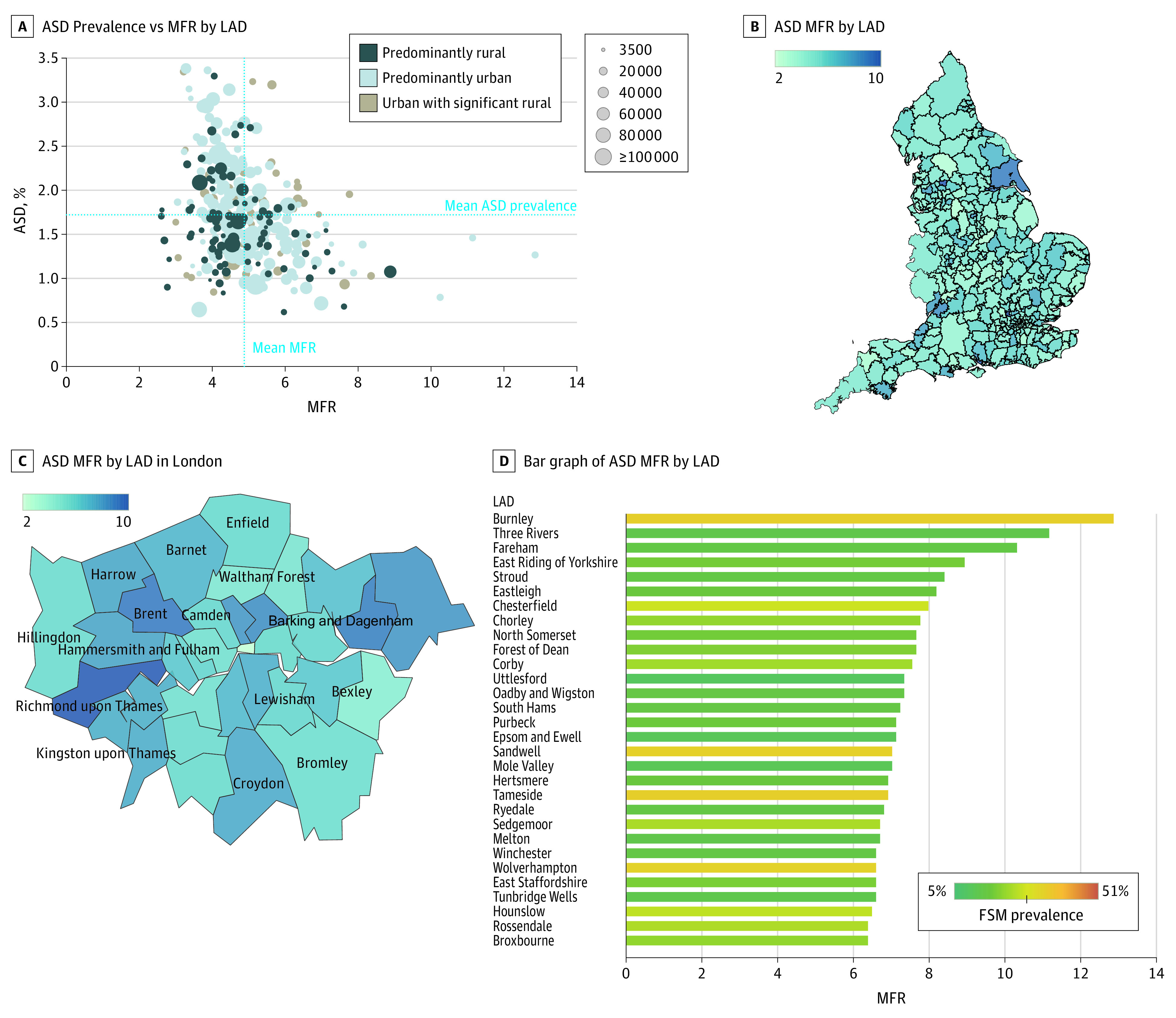
Male-to-Female Ratio (MFR) of Autism Spectrum Disorder (ASD) Prevalence in England Data point sizes represent sample sizes. LAD indicates Local Authority District.

The age- and sex-standardized prevalence of ASD in our national sample was 1.76% (95% CI, 1.75%-1.77%) (male pupils, 2.81% [95% CI, 2.79%-2.83%]; female pupils, 0.65% [95% CI, 0.64%-0.66%]) with an MFR of 4.32:1. Within this group, 58.12% of pupils with ASD had an EHCP (male pupils, 83.66%; female pupils, 16.34%) and 41.88% had support but no EHCP (male pupils, 79.70%; female pupils, 20.30%). Specific details of the standardized prevalence per race/ethnicity, EHCP, and support status are shown in Table 1 and eTable 6 in the Supplement. Standardized prevalence was highest in Black pupils (2.11% [95% CI, 2.06%-2.16%]; MFR, 4.68:1) and lowest in Roma/Irish Travelers (0.85% [95% CI, 0.67%-1.03%]; MFR, 2.84:1). The district with the highest standardized prevalence of ASD was Solihull (3.38% [95% CI, 3.15%-3.61%]; MFR, 3.26:1), and the lowest was the Cotswolds (0.63% [95% CI, 0.46%-0.81%]; MFR, 5.42:1). The MFRs varied across districts from 2.44:1 in Craven to 12.87:1 in Burnsley; other high MFRs were 10.83:1 for Three Rivers and 10.32:1 for Fareham. The districts with the lowest SSR were Newham (0.42), Rushcliffe (0.52), and Forest Heath (0.54), with the highest in Barrow-in-Furness (10.15). Figure 1 shows heat maps for England and London. For all local prevalence estimates, see eTables 4 and 5 in the Supplement. Figure 2 illustrates the MFR distribution of ASD in England and London specifically.

**Table 1.** ASD Prevalence by Sex and Race/Ethnicity

Characteristic by race/ethnicity	Prevalence of ASD			
	All pupils		Male pupils, standardized (95% CI), %	Female pupils, standardized (95% CI), %
	Standardized (95% CI), %	MFR		
Total	1.76 (1.75-1.77)	4.32:1	2.81 (2.79-2.83)	0.65 (0.64-0.66)
Race/ethnicity				
White	1.84 (1.82-1.85)	4.31:1	2.93 (2.91-2.96)	0.68 (0.67-0.69)
Asian	1.06 (1.04-1.09)	4.36:1	1.70 (1.65-1.74)	0.39 (0.37-0.41)
Black	2.11 (2.06-2.16)	4.68:1	3.42 (3.33-3.51)	0.73 (0.69-0.77)
Chinese	1.59 (1.44-1.74)	5.12:1	2.61 (2.35-2.88)	0.51 (0.39-0.63)
Roma/Irish Traveler	0.85 (0.67-1.03)	2.84:1	1.25 (0.95-1.56)	0.44 (0.25-0.62)
Mixed	1.88 (1.83-1.93)	4.29:1	3.00 (2.91-3.09)	0.70 (0.65-0.74)
Other	1.23 (1.16-1.30)	5.05:1	2.02 (1.90-2.14)	0.40 (0.35-0.46)
Unclassified	1.93 (1.82-2.03)	4.18:1	3.05 (2.87-3.24)	0.73 (0.64-0.83)
EHCP				
Total	1.06 (1.05-1.07)	4.91:1	1.72 (1.71-1.74)	0.35 (0.35-0.36)
White	1.05 (1.04-1.06)	10.06:1	1.71 (1.69-1.73)	0.17 (0.16-0.17)
Asian	0.78 (0.75-0.80)	12.40:1	1.24 (1.20-1.27)	0.10 (0.09-0.11)
Black	1.65 (1.60-1.69)	15.71:1	2.67 (2.59-2.75)	0.17 (0.15-0.19)
Chinese	1.17 (1.04-1.30)	13.79:1	1.93 (1.70-2.16)	0.14 (0.08-0.20)
Roma/Irish Traveler	0.71 (0.53-0.89)	10.70:1	1.07 (0.77-1.37)	0.10 (0.05-0.14)
Mixed	1.19 (1.15-1.23)	6.29:1	1.95 (1.88-2.02)	0.31 (0.28-0.34)
Other	0.90 (0.84-0.96)	11.46:1	1.49 (1.38-1.59)	0.13 (0.09-0.16)
Unclassified	1.26 (1.16-1.34)	6.58:1	2.04 (1.88-2.20)	0.31 (0.25-0.37)
SEND support				
Total	0.70 (0.69-0.71)	3.72:1	1.08 (1.07-1.09)	0.29 (0.29-0.30)
White	0.79 (0.78-0.80)	3.70:1	1.22 (1.21-1.23)	0.33 (0.33-0.34)
Asian	0.28 (0.27-0.30)	4.60:1	0.46 (0.44-0.48)	0.10 (0.09-0.11)
Black	0.47 (0.44-0.49)	4.41:1	0.75 (0.71-0.79)	0.17 (0.15-0.19)
Chinese	0.42 (0.34-0.49)	4.86:1	0.68 (0.55-0.81)	0.14 (0.08-0.20)
Roma/Irish Traveler	0.14 (0.10-0.18)	1.90:1	0.19 (0.13-0.25)	0.10 (0.05-0.14)
Mixed	0.69 (0.66-0.72)	3.39:1	1.05 (1.00-1.11)	0.31 (0.28-0.34)
Other	0.33 (0.30-0.37)	4.08:1	0.53 (0.47-0.59)	0.13 (0.09-0.16)
Unclassified	0.67 (0.61-0.74)	3.26:1	1.01 (0.91-1.12)	0.31 (0.25-0.37)

Abbreviations: ASD, autism spectrum disorder; EHCP, Education, Health and Care Plan; MFR, male-to-female ratio; SEND, Special Educational Needs and Disability.

### Poisson Regression Analysis Results

Autism spectrum disorder was almost 4 and a half times more likely in male pupils than female pupils (aPR, 4.39 [95% CI, 4.33-4.45]). In this same analysis, pupils aged 10 to 14 years (aPR, 1.19 [95% CI, 1.18-1.21]) and 15 to 19 years (aPR, 1.21 [95% CI, 1.19-1.23]) were more likely to have ASD than those aged 5 to 9 years. Pupils from a Roma/Irish Traveler background (aPR, 0.42 [95% CI, 0.36-0.48]), Asian background (aPR, 0.83 [95% CI, 0.81-0.85]), and any other racial/ethnic group (aPR, 0.92 [95% CI, 0.87-0.97]) were less likely to have ASD compared with White pupils. In contrast, pupils from Chinese (aPR, 1.38 [95% CI, 1.26-1.50]), Black (aPR, 1.26 [95% CI, 1.23-1.29]), and unclassified (aPR, 1.09 [95% CI, 1.03-1.15]) backgrounds were more likely to have ASD. Autism spectrum disorder was also more likely in pupils who had ever been eligible for the FSM program (aPR, 1.61 [95% CI, 1.59-1.63]). Pupils whose first language was not English were less likely to have an ASD diagnosis (aPR, 0.64 [95% CI, 0.63-0.65]). Details are shown in Table 2 and eFigures 1 to 5 in the Supplement.

**Table 2.** Poisson Regression Model for aPR With a Robust Error Variance Using Multiple Imputation Chained Equations for Missing Data

Characteristic	aPR (95% CI)	*P* value
**ASD status**		
Age, y		
5-9	1 [Reference]	NA
10-14	1.19 (1.18-1.21)	<.001
15-19	1.21 (1.19-1.23)	<.001
Sex		
Female	1 [Reference]	NA
Male	4.39 (4.33-4.45)	<.001
Race/ethnicity		
White	1 [Reference]	NA
Asian	0.83 (0.81-0.85)	<.001
Black	1.26 (1.23-1.29)	<.001
Chinese	1.38 (1.26-1.50)	<.001
Roma/Irish Traveler	0.42 (0.36-0.48)	<.001
Mixed	1.02 (1.00-1.05)	.055
Other	0.92 (0.87-0.97)	.001
Unclassified	1.09 (1.03-1.15)	.001
Eligible for FSM ever		
No	1 [Reference]	NA
Yes	1.61 (1.59-1.63)	<.001
Language at home		
English	1 [Reference]	NA
Other	0.64 (0.63-0.65)	<.001
Unknown	0.77 (0.67-0.89)	<.001
**ASD EHCP**		
Age, y		
5-9	1 [Reference]	NA
10-14	1.18 (1.16-1.20)	<.001
15-19	1.44 (1.40-1.47)	<.001
Sex		
Female	1 [Reference]	NA
Male	4.94 (4.85-5.04)	<.001
Race/ethnicity		
White	1 [Reference]	NA
Asian	1.02 (0.99-1.06)	.14
Black	1.68 (1.64-1.73)	<.001
Chinese	1.73 (1.56-1.92)	<.001
Roma/Irish Traveler	0.53 (0.45-0.63)	<.001
Mixed	1.14 (1.11-1.18)	<.001
Other	1.11 (1.05-1.19)	.001
Unclassified	1.26 (1.18-1.34)	<.001
Eligible for FSM ever		
No	1 [Reference]	NA
Yes	1.71 (1.68-1.74)	<.001
Language at home		
English	1 [Reference]	NA
Other	0.70 (0.68-0.72)	<.001
Unclassified	0.64 (0.53-0.78)	<.001
**ASD support**		
Age, y		
5-9	1 [Reference]	NA
10-14	1.20 (1.18-1.22)	<.001
15-19	0.92 (0.89-0.95)	<.001
Sex		
Female	1 [Reference]	NA
Male	3.77 (3.69-3.85)	<.001
Race/ethnicity		
White	1 [Reference]	NA
Asian	0.58 (0.55-0.60)	<.001
Black	0.72 (0.68-0.75)	<.001
Chinese	0.93 (0.79-1.10)	.39
Roma/Irish Traveler	0.27 (0.20-0.35)	<.001
Mixed	0.88 (0.85-0.91)	<.001
Other	0.66 (0.60-0.73)	<.001
Unclassified	0.89 (0.81-0.97)	.006
Eligible for FSM ever		
No	1 [Reference]	NA
Yes	1.49 (1.46-1.51)	<.001
Language at home		
English	1 [Reference]	NA
Other	0.54 (0.52-0.56)	<.001
Unclassified	1.01 (0.82-1.25)	.91
**ASD and secondary SEND**		
Age, y		
5-9	1 [Reference]	NA
10-14	1.11 (1.09-1.13)	<.001
15-19	1.01 (1.98-1.03)	.48
Sex		
Female	1 [Reference]	NA
Male	4.47 (4.38-4.56)	<.001
Race/ethnicity		
White	1 [Reference]	NA
Asian	0.82 (0.79-0.85)	<.001
Black	1.22 (1.18-1.26)	<.001
Chinese	1.41 (1.25-1.58)	<.001
Roma/Irish Traveler	0.36 (0.29-0.44)	<.001
Mixed	1.00 (0.96-1.03)	.89
Other	0.94 (0.88-1.01)	.11
Unclassified	1.12 (1.04-1.20)	.002
Eligible for FSM ever		
No	1 [Reference]	NA
Yes	1.43 (1.40-1.45)	<.001
Language at home		
English	1 [Reference]	NA
Other	0.62 (0.60-0.63)	<.001
Unclassified	0.99 (0.83-1.18)	.90

Abbreviations: aPR, adjusted prevalence ratio; ASD, autism spectrum disorder; EHCP, Education, Health and Care Plan; FSM, Free School Meals program; NA, not applicable; SEND, Special Educational Needs and Disability.

Older pupils were more likely to have an EHCP (aPR for 10-14 years of age, 1.18 [95% CI, 1.16-1.20]; aPR for 15-19 years of age, 1.44 [95% CI, 1.40-1.47]). Male pupils were more likely than female pupils to receive an ASD EHCP (aPR, 4.94 [95% CI, 4.85-5.04]). Black (aPR, 1.68 [95% CI, 1.64-1.73]) and Chinese (aPR, 1.73 [95% CI, 1.56-1.92]) pupils were more likely to have an EHCP than White pupils. Pupils who were ever eligible for the FSM program were also more likely to receive an ASD EHCP (aPR, 1.71 [95% CI, 1.68-1.74]). In contrast, Roma/Irish Traveler pupils were less likely to receive an ASD EHCP (aPR, 0.53 [95% CI, 0.45-0.63]). When investigating the relationship between receiving ASD SEND support and race/ethnicity, non-White pupils were less likely to receive support when compared with White pupils.

In our 1-way sensitivity analysis comparing both negative missingness of FSM (using liberal assumptions) and a listwise deletion model of FSM (using conservative assumptions), we observed outcomes similar to those from our multiple imputation by chained equation models (eTables 2 and 3 in the Supplement), increasing confidence in our imputation method. In the crude model, there was no loss due to missing data. The complete case analysis had complete data for 93.81% (n = 6 611 261).

### Mediation Analysis

Our mediation analysis compared the association of social disadvantage (using the FSM program as a proxy) with ASD, with White pupils as the reference group. Our results showed an indirect effect of race/ethnicity with ASD status through FSM. Black pupils and those of mixed race/ethnicity showed notably higher effect sizes (standardized mediation coefficient [SMC], 0.018 [*P* < .001] with 12.41% of effects mediated and 0.010 [*P* < .001] with 12.05% of effects mediated, respectively). In other words, 12.41% and 12.05% of the increased ASD prevalence in these groups can be explained by the relative social disadvantage associated with their race/ethnicity when compared with White pupils. In the case of first language spoken as a mediator of ASD status, we obtained inconsistent mediation (SMC range for race/ethnicity categories, −0.007 to 0.001; SMC for first language other than English, −0.132). For all details, see Table 3; for models used, see eFigures 7 and 8 in the Supplement.

**Table 3.** Mediation Effects of FSM and Language Spoken at Home on Race/Ethnicity and ASD Status

Characteristic	Standardized coefficients	Mediation proportion, %	SE	Estimate/SE	*P* value for bootstrap
Language spoken at home by race/ethnicity					
White	1 [Reference]	NA	NA	NA	NA
Asian	0.525	NA	0	1544.40	<.001
Black	0.246	NA	0	648.98	<.001
Chinese	0.114	NA	0	274.83	<.001
Roma/Irish Traveler	−0.004	NA	0	−112.54	<.001
Mixed	0.095	NA	0	209.38	<.001
Other	0.249	NA	0	595.12	<.001
Unclassified	0.053	NA	0	118.96	<.001
FSM by race/ethnicity					
White	1 [Reference]	NA	NA	NA	NA
Asian	0.012	NA	0.001	22.89	<.001
Black	0.145	NA	0	301.27	<.001
Chinese	−0.024	NA	0.001	−40.22	<.001
Roma/Irish Traveler	0.007	NA	0	182.97	<.001
Mixed	0.083	NA	0	169.01	<.001
Other	0.053	NA	0	108.71	<.001
Unclassified	0.019	NA	0.001	37.44	<.001
ASD status					
Language spoken at home	−0.130	NA	0.003	−46.40	<.001
FSM	0.124	NA	0.002	77.98	<.001
Race/ethnicity					
White	1 [Reference]	NA	NA	NA	NA
Asian	0.010	NA	0.002	5.01	<.001
Black	0.032	NA	0.001	24.40	<.001
Chinese	0.014	NA	0.001	11.29	<.001
Roma/Irish Traveler	0.011	NA	0	123.50	<.001
Mixed	0.007	NA	0.001	5.68	<.001
Other	0.009	NA	0.001	6.36	<.001
Unclassified	0.009	NA	0.001	7.78	<.001
Indirect effects from any other racial/ethnic group to ASD status					
Any other racial/ethnic group > FSM > ASD	0.007	13.21	0	63.34	<.001
Any other racial/ethnic group > language spoken at home > ASD	−0.032	i	0.001	−46.25	<.001
Indirect effects from Asian to ASD status					
Asian > FSM > ASD	0.001	8.33	0	21.97	<.001
Asian > language spoken at home > ASD	−0.068	i	0.001	−46.37	<.001
Indirect effects from Black to ASD status					
Black > FSM > ASD	0.018	12.41	0	75.43	<.001
Black > language spoken at home > ASD	−0.032	i	0.001	−46.27	<.001
Indirect effects from Chinese to ASD status					
Chinese > FSM > ASD	−0.003	12.50	0	−35.73	<.001
Chinese > language spoken at home > ASD	−0.015	i	0	−45.74	<.001
Indirect effects from mixed race/ethnicity to ASD status					
Mixed race/ethnicity > FSM > ASD	0.010	12.05	0	7.75	<.001
Mixed race/ethnicity > language spoken at home > ASD	−0.012	i	0	−45.27	<.001
Indirect effects from unclassified race/ethnicity to ASD status					
Unclassified race/ethnicity > FSM > ASD	0.002	10.53	0	33.74	<.001
Unclassified race/ethnicity > language spoken at home > ASD	−0.007	i	0	−43.21	<.001
Indirect effects from Roma/Irish Traveler to ASD status					
Roma/Irish Traveler > FSM > ASD	0.001	14.29	0	71.62	<.001
Roma/Irish Traveler > language spoken at home > ASD	0.001	i	0	42.89	<.001

Abbreviations: ASD, autism spectrum disorder; FSM, Free School Meals program; i, inconsistent mediation; NA, not applicable.

## Discussion

This study reports formally recorded ASD prevalence in English pupils aged 5 to 19 years using a total school population sample, providing evidence of consistent prevalence differences among different racial/ethnic groups. The reported English standardized prevalence of ASD in our sample was 1.76% (95% CI, 1.75%-1.77%), with male pupils showing a prevalence of 2.81% (95% CI, 2.79%-2.83%) and female pupils, 0.65% (95% CI, 0.64%-0.66%), with an MFR of 4.32:1. This is a considerable increase from previously reported prevalence estimates in England that described a 1.57% autism prevalence in 2009 using a school SEND registry and diagnostic survey methods.^15^ Strikingly, the standardized prevalence of ASD was highest in Black pupils (2.11% [95% CI, 2.06%-2.16%]) and lowest in Roma/Irish Traveler pupils (0.85% [95% CI, 0.67%-1.03%]), showing consistent differences in autism prevalence according to race/ethnicity. The standardized prevalence of ASD spread across England was varied, with several possible reasons. First, diagnoses are typically made in the National Health Service, and although diagnostic services tend to be robust across the UK, standardized procedures, protocols, and diagnostic tools or instruments used by clinical teams are inconsistent. Second, variability exists in the provision of education and special educational support across England. Third, significant differences exist in the thresholds for accessing SEND support or an EHCP. Finally, real differences in prevalence might exist between different areas.^6^ Another striking finding was the large variance in MFR across districts (2.44:1 in Craven to 12.87:1 in Burnsley), which could indicate shortcomings of the current diagnostic process for female pupils with ASD.

Our results further show that Chinese and Black pupils were 38% and 26% more likely to be recorded with ASD in the English educational system than White pupils (aPR, 1.38 [95% CI, 1.26-1.50] and 1.26 [95% CI, 1.23-1.29], respectively). Pupils from a Roma/Irish Traveler background were almost 60% less likely to be recorded with ASD compared with White pupils (aPR, 0.42 [95% CI, 0.36-0.48]). Pupils whose first language was not English were less likely to have an ASD diagnosis (aPR, 0.64 [95% CI, 0.63-0.65]) in the English education system. Our findings show that pupils facing social disadvantage were more likely to have ASD (aPR, 1.61 [95% CI, 1.59-1.63]). Exploring this issue further, we found that racial/ethnic differences in ASD were mediated through socioeconomic disadvantage. The greatest effect was found among Black pupils (standardized mediation coefficient, 0.018; *P* < .001), with 12.41% of the increased prevalence of ASD among Black pupils being explained by social disadvantage. The interaction between ASD status and social disadvantage can be 2-fold: (1) children from socially disadvantaged families may be at higher odds of developing ASD, or (2) having a child with ASD can increase the risk of a family experiencing poverty. The indirect effect of race/ethnicity on ASD status through first language spoken was inconsistent in our analysis. Although the estimates obtained for race/ethnicity were consistently larger than zero (SMC range, −0.004 to 0.525), this is opposed by the estimate obtained when English was not the first language spoken (SMC, −0.130). These statistics support our hypothesis that although some racial/ethnic groups are more likely to receive an ASD diagnosis or support, this might be counterbalanced by barriers in accessing SEND support in families who speak other languages at home, such as Asian families (73.77% of whom speak other first languages) and Black families (43.68% of whom speak other first languages). This is an important issue considering the social dimensions of the core features of ASD and how they are recognized in bilingual pupils. Other issues shown in our findings are sex differences and the large regional variation in the MFR observed across English districts, with Burnley (12.87:1), Three Rivers (10.83:1), and Fareham (10.32:1) having the highest MFRs in England. The same can be said to the SSR that we report, with Newham (0.42), Rushcliffe (0.52), and Forest Heath (0.54) having the lowest ratios. These rates indicate approximately 1 pupil with EHCP per 2 pupils with support in these districts. When comparing these rates with the national mean SSR for pupils with ASD, it becomes evident that EHCPs are underprovided in some districts (eTable 4 in the Supplement).

### Limitations

This study has a number of limitations. First, the proportion of pupils aged 3 to 18 years attending state-funded schools in England is 93%, with 7% enrolled in independent schools or in alternative arrangements such as home schooling.^22^ Another limitation is that pupils from certain racial/ethnic backgrounds (such as Roma/Irish Traveler) tend to leave school prematurely, with some evidence demonstrating that approximately half leave school by 16 years of age.^23^ Considering that we used World Health Organization–validated direct standardization methods with British census data, this issue has been addressed to the best of our ability given the resources available. A different hypothesis regarding the low ASD prevalence figures found among the Roma/Irish Traveler community might involve questioning whether diagnostic recognition of ASD symptoms (such as communication and repetitive behaviors) in the English educational system adversely affects this subgroup, and whether it is culturally appropiate.^23,24,25^ Although complex challenges exist in construing prevalence estimates with administrative data, we assessed health service and educational system use, and our findings reflect actual numbers of individuals receiving services in schools by locality.^26^ Furthermore, the NPD does not account for pupils with subclinical ASD or those who do not meet service thresholds to receive support or EHCPs at school.^15^ Further research should explore the reasons for this, but more importantly should focus on the dynamic between an ASD diagnosis and the school system.

## Conclusions

Our results highlighting higher ASD prevalence in pupils of racial/ethnic minority groups are in line with a broader literature of large population-based studies using health and education registry data that have described an increased ASD prevalence in pupils of racial/ethnic minority and immigrant backgrounds both in European and Nordic countries.^9,27^ When comparing our findings with US evidence, they are in line with the findings of a population study by Becerra et al that found higher ASD rates in immigrants with foreign-born mothers,^28^ whereas they contrast with the findings of Durkin et al that the Black community and people with a lower socioeconomic status have notably lower ASD rates.^29^ Our findings also challenge evidence from the Centers for Disease Control and Prevention–Autism and Developmental Disabilities Monitoring (ADDM) Network in the US that showed wide variation between sites in 2016^30^; for example, Colorado reported a prevalence of 1.31% (95% CI, 1.21%-1.43%), whereas the prevalence in New Jersey was 3.14% (95% CI, 2.95%-3.33%), and Colorado reported no differences according to race/ethnicity. Higher prevalence in racial/ethnic minority groups has also been reported for other neurodevelopmental conditions, such as schizophrenia, with racial/ethnic minority and immigration status found to be associated with higher risk of developing psychosis.^31^ The large genetic overlap between these 2 neurodevelopmental conditions should be taken into consideration when investigating possible links between immigration and racial/ethnic minority and ASD status.^32,33,34,35^ We also believe that it is crucial to assess the intersection of ASD policy and public health policy as well as their assumptions about racial/ethnic minority groups, immigrants, and other typically underserved populations.^36^ Our results challenge us to better understand which pupils receive an ASD diagnosis, when they receive it, what support is provided to them, and, most importantly, to what degree social determinants of health, immigration, and race/ethnicity affect ASD status.^36^

## Notes

Supplement. **eMethods.** National Pupil Database and Study Components **eTable 1.** Descriptive Statistics **eTable 2.** Sensitivity Analysis for Adjusted Prevalence Ratios Log-Binomial and Poisson Regression With Robust Error Variance in Missing Information and Complete Case Analysis Model for Autism Spectrum Disorder Status as Outcome **eTable 3.** Sensitivity Analysis for Adjusted Prevalence Ratios Poisson Regression With MICE Estimation Using Autism Spectrum Disorder Status as Outcome and Including Local Authority Districts Decile Index of Multiple Deprivation **eTable 4.** Age and Sex Standardized Prevalence of Autism Spectrum Disorder by Local Authority District **eTable 5.** Age- and Sex-Specified Prevalence Rates for SEND Statement and Support by Local Area District **eTable 6.** ASD Prevalence by LAD and MFR **eFigure 1.** SPR Spread of Autism Spectrum Disorder by Sex Compared Learning Difficulties by LAD **eFigure 2.** Variability of All Other SEND Categories by LAD **eFigure 3.** FSM Prevalence in England and London **eFigure 4.** Standardized Prevalence of Autism Spectrum Disorder by LAD, Male-to-Female-Ratio and Sex and Reporting by Support and EHCP **eFigure 5.** Ratio of SEND Statements vs SEND Support in England **eFigure 6.** Hypothesized Model of the Mediation Analysis **eFigure 7.** Final Model of the Mediation Analysis **eFigure 8.** Indirect Effects Results
