# Supplementary material for: Association of Race/Ethnicity and Social Disadvantage With Autism Prevalence in 7 Million School Children in England
Source: JAMA Pediatr. 2021 Mar 29;175(6):e210054. doi: 10.1001/jamapediatrics.2021.0054 (PMC8008434; doi:10.1001/jamapediatrics.2021.0054)
Supplement: Supplement. — eMethods. National Pupil Database and Study Components eTable 1. Descriptive Statistics eTable 2. Sensitivity Analysis for Adjusted Prevalence Ratios Log-Binomial and Poisson Regression With Robust Error Variance in Missing Information and Complete Case Analysis Model for Autism Spectrum Disorder Status as Outcome eTable 3. Sensitivity Analysis for Adjusted Prevalence Ratios Poisson Regression With MICE Estimation Using Autism Spectrum Disorder Status as Outcome and Including Local Authority Districts Decile Index of Multiple Deprivation eTable 4. Age and Sex Standardized Prevalence of Autism Spectrum Disorder by Local Authority District eTable 5. Age- and Sex-Specified Prevalence Rates for SEND Statement and Support by Local Area District eTable 6. ASD Prevalence by LAD and MFR eFigure 1. SPR Spread of Autism Spectrum Disorder by Sex Compared Learning Difficulties by LAD eFigure 2. Variability of All Other SEND Categories by LAD eFigure 3. FSM Prevalence in England and London eFigure 4. Standardized Prevalence of Autism Spectrum Disorder by LAD, Male-to-Female-Ratio and Sex and Reporting by Support and EHCP eFigure 5. Ratio of SEND Statements vs SEND Support in England eFigure 6. Hypothesized Model of the Mediation Analysis eFigure 7. Final Model of the Mediation Analysis eFigure 8. Indirect Effects Results [file jamapediatr-e210054-s001.pdf]

Supplemental Online Content

Roman-Urrestarazu A, van Kessel R, Allison C, Matthews FE, Brayne C, Baron-Cohen S. Association of race/ethnicity and social disadvantage with autism prevalence in 7 million school children in England. *JAMA Pediatr.* Published online March 29, 2021. doi:10.1001/jamapediatrics.2021.0054

eMethods. National Pupil Database and Study Components
eTable 1. Descriptive Statistics
eTable 2. Sensitivity Analysis for Adjusted Prevalence Ratios Log-Binomial and Poisson Regression With Robust Error Variance in Missing Information and Complete Case Analysis Model for Autism Spectrum Disorder Status as Outcome
eTable 3. Sensitivity Analysis for Adjusted Prevalence Ratios Poisson Regression With MICE Estimation Using Autism Spectrum Disorder Status as Outcome and Including Local Authority Districts Decile Index of Multiple Deprivation
eTable 4. Age and Sex Standardized Prevalence of Autism Spectrum Disorder by Local Authority District
eTable 5. Age- and Sex-Specified Prevalence Rates for SEND Statement and Support by Local Area District
eTable 6. ASD Prevalence by LAD and MFR
eFigure 1. SPR Spread of Autism Spectrum Disorder by Sex Compared Learning Difficulties by LAD
eFigure 2. Variability of All Other SEND Categories by LAD
eFigure 3. FSM Prevalence in England and London
eFigure 4. Standardized Prevalence of Autism Spectrum Disorder by LAD, Male-to-Female-Ratio and Sex and Reporting by Support and EHCP
eFigure 5. Ratio of SEND Statements vs SEND Support in England
eFigure 6. Hypothesized Model of the Mediation Analysis
eFigure 7. Final Model of the Mediation Analysis
eFigure 8. Indirect Effects Results

This supplementary material has been provided by the authors to give readers additional information about their work.

©2021 Roman-Urrestarazu A et al. *JAMA Pediatrics*.

1. *The National Pupil Database*

The NPD includes data from different sources including (a) The School Census - Pupil Level Annual Schools Census (PLASC), (b) The Pupil Referral Unit (PRU) Census and (c) Alternative Provision all from ages 2-19. The School Census is routinely carried out three times a year and provides the DfE with both pupil and school-level data from (a) Nursery (maintained and direct grant nursery schools), (b) Primary (primary schools and academies), (c) Secondary (secondary schools and academies), (d) City Technology Colleges and (e) Special (maintained and non-maintained special schools, hospital special schools and academies). Full-time education is compulsory between 5 to 18 years in England, reason why we included pupils aged 5-19 years.

2. *Geographies Used in Analysis*

As the structure of local government in England is not uniform, there are currently four principal types of district-level subdivisions. There is a total of 326 LADs made up of 36 metropolitan boroughs, 32 London boroughs, 201 non-metropolitan districts, 55 unitary authorities, as well as the City of London and the Isles of Scilly which are also districts, but do not correspond to any of these categories.

3. *Descriptive Statistics and ethnic composition of different categories*

Individuals were categorised into three age groups (5-9, 10-14, and 15-19 years old). Sex was coded as either female or male. NPD has eight major categorisations of ethnicity including (1) "any other ethnic group" (AOEG) which comprises Afghanistani, Arab, Egyptian, Filipino, Iranian, Iraqi, Japanese, Korean, Kurdish, Latin American, Lebanese, Libyan, Malay, Moroccan, Other ethnic group, Any other ethnic group, Polynesian, Thai, Vietnamese and Yemeni people; (2) "Asian" (ASIA) and this category includes African Asian, Bangladeshi Indian, Kashmiri, Other Kashmiri, Pakistani Mirpuri, Pakistani, Nepali, Other Pakistani, Other Asian, Any Other Asian background, Pakistani, Sri Lankan Tamil, Sri Lankan Sinhalese and Sri Lankan Other people; "(3) black" (BLAC) which includes Black African, Caribbean and Any other black background, (4) "Chinese" (CHIN) which includes Hong Kong Chinese, Chinese, Malaysian Chinese, Other Chinese, Singaporean Chinese and Taiwanese people; (5) "mixed" (MIXD) which includes all options of mixed ethnic backgrounds; (6) "unclassified" (UNCL) which includes people who refused to answer this question, (7) "white" (WHIT) which includes Any other white background and White British and finally (8) "Roma/Irish

©2021 Roman-Urrestarazu A et al. *JAMA Pediatrics*.

Traveller" (ROM/TRAV). In Table 1 we present all the detailed descriptive statistics of our sample.

4. *SEND Categories*

Currently the PLASC includes 14 SEND categories: (1) Unclassified (UNCL) (not reported in descriptive statistics), (2) Specific Learning Difficulty (SPLD), (3) Moderate Learning Difficulty (MLD), (4) Severe Learning Difficulty (SLD), (5) Profound and Multiple Learning Difficulty (PMLD), (6) Speech, Language and Communication Needs (SLCN), (7) Hearing Impairment (HI), (8) Visual Impairment (VI), (9) Multi-Sensory Impairment (MSI), (10) Physical Disability (PD), (11) Autism (ASD), (12) Other Difficulty/Disability (OTH), (13) Social, emotional and mental health, (SEMH), and (14) SEND support but No Specialist Assessment (NSA). The PLASC allows up to two different SEND categories in the same pupil, so we defined autism as any pupil presenting a SEND as a primary or secondary category. We use descriptive statistics for the other SEND categories while also reporting autism comorbidities and co-conditions. In the case of learning difficulties (LD) there are four different categories defined by level of severity, so we report a compound variable and the specific four severity variables. We did the same for sensory SEND. Young people and parents of children who have an EHCP have the right to request a Personal Budget, which is funding provided by the local authority to deliver services set out in an EHCP.

Note that the British definition of Learning Difficulties closely resembles intellectual disability and specific learning disorder in the ICD-11 and DSM-5 respectively. The main difference is that ICD-11 and DSM-5 entries refer to someone who has an IQ below 70 with global affectation, while the British definition used in this article refers to specific challenges in learning such as Dyslexia, Dyspraxia, Dyscalculia and Dysgraphia.

©2021 Roman-Urrestarazu A et al. *JAMA Pediatrics*.

In Figure 1 we present the SPR spread of autism by sex and compare this to learning difficulties by LAD. As one can observe the spread is much larger for learning difficulties and autistic boys, with women having a more stable reported prevalence that still shows variability across LAD.

In Figure 2 we present the variability of all other SEND categories by LAD.

5. *FSM and Socio-economic Disadvantage*

Under the universal FSM, every child in year 1 and year 2 in England is universally entitled to a free hot lunch if they claim eligibility. If they are older, children are eligible for FSM if their parents (1) claim state benefits, (2) are paid income support, (3) are on jobseeker's allowance, (4) have employment and support allowance, (5) have pension credit, and or child tax credit, or (6) have working tax credit and/or claim universal credit. Pupils who are child asylum seekers are also entitled to FSM.

6. *Statistical Model and Sensitivity Analysis*

To describe effect size we used validated guidelines that describe small effect sizes coefficients below 0.01, medium effects would be between 0.01 and 0.09, and large effects would be higher than 0.25. In our MICE model, for replicable SE estimates, the required number of imputations increases quadratically with the fraction of missing information, so we used the quadratic function to calculate the number of imputations needed for the analyses, which was 10.[1]

We also performed an analysis including local authority districts deciles of the English index of multiple deprivation (IMD) from 2019 in order to also analyse the effect of socio-spatial deprivation on autism status in the English education system using the same MICE Poisson regression model of our main results section. IMD are widely used datasets within the UK to classify the relative deprivation (essentially a measure of poverty) of small areas. Multiple components of deprivation are weighted with different strengths and compiled into a single

score of deprivation. The use of IMDs in social analysis aims to balance the desire for a single number describing the concept of deprivation in a place and the recognition that deprivation has many interacting components. IMDs may be an improvement over simpler measures of deprivation such as low average household disposable income because they capture variables such as the advantage of access to a good school and the disadvantage of exposure to high levels of air pollution. A potential disadvantage is that the choice of components and the weighting of those components in the construction of the overall multiple deprivation score is unavoidably subjective. In the current English Indices of Deprivation 2019 (IoD2019) seven domains of deprivation are considered and weighted as follows:

a. Income. (22.5%)
b. Employment. (22.5%)
c. Education. (13.5%)
d. Health. (13.5%)
e. Crime. (9.3%)
f. Barriers to Housing and Services. (9.3%)
g. Living Environment. (9.3%)

These domains each have multiple components. For example, the Barriers to Housing and Services considers seven components including levels of household overcrowding, homelessness, housing affordability, and the distance by road to four types of key amenity (post office, primary school, supermarket, and GP surgery). We observed that our model is robust to the inclusion of the IMD and maintains similar outcomes and results even after adding this variable which shows that in areas of socio-spatial deprivation children are 29% more likely to have a recognised autism diagnosis at school. These results support our assessment of social deprivation on autism status and furthermore it complements our family level measure of socio-economic deprivation (claimed eligibility of free school meals) by adding a widely validated geographical measure such as the index of multiple deprivation.

7. *Autism and Ethnic Minorities*

Our results are in line with other literature describing differences in prevalence in ethnic minorities. Keen and colleagues for example reported an increased likelihood for black mothers

©2021 Roman-Urrestarazu A et al. *JAMA Pediatrics*.

of having an autistic child in two centres in the UK compared to white mothers (N=428; relative risk [RR] 8.28; 95% CI 5.41–12.7 and RR 3.84; 95% CI 2.93–5.02).[2] These findings were subsequently replicated by Magnusson and colleagues, in their investigation in Sweden, where they reported an increased odds of low-functioning autism (N=589 306; OR 1.5; 95% CI 1.3 – 1.7) in children of migrant parents, which was even higher if the migration happened around pregnancy (OR 2.3; 95% CI 1.7 – 3.0).[3] Also in support of our work is the recent work by Winter et al that found that between 1998 and 2018 found a 612% increase in autism incidence that did not rise uniformly across sociodemographic groups[4]. This works shows how by 2018, children of Black and Asian mothers were diagnosed at higher rates than children of non-Hispanic White mothers[4]. Furthermore, among children of non-Hispanic White and Asian mothers, children of lower SES were diagnosed at higher rates than children of higher SES [4]. Finally, Becerra and colleagues also reported an increased likelihood of autism diagnosis in black, foreign-born children compared to white, USA-born children (N= 1 626 354; RR 1.76; 95% CI 1.41 – 2.18).[5] Furthermore we also describe differences in sex in line with the work of Hull and colleagues[6] indicate that there may not only be sex differences in phenotype but that these differences may be dependent on the age of the individual. This could also contribute to the explanation as to why younger children seem to receive less autism diagnoses, even though awareness of autism has increased recently. Another element that can contribute to explaining these differences is the notion that autistic females have a higher tendency to camouflage[7], which can further complicate recognising autistic traits in females.

8. Disclosure Threshold

Throughout this project, the disclosure threshold was set to 10 (i.e. N=10). This value is used by the Office for National Statistics (ONS) for England and has subsequently been adopted by a number of other Safe Settings and government departments.

9. Mediation models used

The mediation models used are shown in Figures 6, 7, and 8.

10. Results Analysis

While our results were interpreted from an epidemiological perspective, an alternative interpretation should consider possible diagnostic biases that might account for the different prevalence of autism found among black and other ethnic communities. It is also important to evaluate the relationship between EHCPs and support in autistic pupils across districts, and in other SEND categories, since young people and parents of pupils who have EHCPs have the right to request a Personal Budget and scant research has been conducted looking at austerity and its associations to EHCP and SEND support across districts. Proof of this is the high SPR of both Social Emotional and Mental Health SEND (3.11) and Speech, Language and Communication Needs (2.90), which arguably tend to operate more as a working diagnosis and might harbour pupils with different SEND, including autistic pupils. Considering our autism prevalence estimates, this is a crucial issue in relation to access to early diagnostic services and specialised SEND provision in school. This expectation is reflected in our dataset, where more autistic pupils with EHCP (N=69636; 58.12% of all autistic pupils) were recorded than pupils with support (N=50185; 41.88%).

Important to note is how health system differences between the USA and the UK might bias American estimates considering that previous analyses from the ADDM Network have shown a positive association between neighbourhood socioeconomic status (SES) and autism prevalence, which suggests autism might be more readily identified in high-SES communities or among populations with good health care cover[8]. Considering that universal health coverage is a pillar of the NHS as a single payer system, we believe that American prevalence data might not be able to capture true differences by ethnicity or immigration status, taking into account that immigrants might face serious hurdles to adequate and opportune health services in the USA as shown in the work of Fountain et al[9]. This aligns with our prevalence dispersion and

©2021 Roman-Urrestarazu A et al. *JAMA Pediatrics*.

supports the need for further research into possible ethnic differences in autism prevalence. According to our data, children aged 10-14 and 15-19 are 19% and 21% more likely of being recorded with autism respectively, while children who speak another language at home than English are 30% less likely of being recorded with autism. We are aware that these findings might be an expression of phenotypic differences, but we emphasize that they might also be the result of a higher salience of autism symptoms and or service provision issues in minority ethnic groups, which should also be addressed in future research[10]. Another factor that needs to be considered is that language spoken appears to be a barrier to obtaining an autism diagnosis. Zuckerman et al also report this finding in Latino communities in the USA, who often do not disclose developmental problems due to concerns about healthcare provider communication and access to language services to help interpret their needs[11]. The outlier to this would-be Chinese pupils, who have the highest percentage of speaking other first languages (70.98%), but autism is still more likely to be recognised in this ethnic group (aPR: 1.38; 95% CI 1.26 – 1.50, p< 0.001). Furthermore, in support of our results we can argue that maternal birth abroad has been described in a meta-analysis by Gardener et al as the main prenatal factor linking autism status with ethnicity (OR 1.28), supporting our finding that parental immigration and ethnicity, might be involved in higher autism prevalence in ethnic minorities in the UK.[12]

©2021 Roman-Urrestarazu A et al. *JAMA Pediatrics*.

Table 1. Descriptive Statistics

			School Children No SEND			Autism			Autism and Learning Difficulty			School Children Any Other SEND			Total School Children		
			n	% From Total	95% CI	n	% From Total	95% CI	n	% From Total	95% CI	n	% From Total	95% CI	n	% From Total	95% CI
Sex distribution	Male		3095530	51.79%	51.74 – 51.82%	80788	82,00%	81.78 – 82.22%	17466	80.64%	80.11 – 81.16%	336644	35.44%	35.35 – 35.54%	3593497	50.99%	50.95 – 51.03%
	Female		2882122	48.21%	48.17 – 48.26%	17373	17.99%	17.78 – 18.22%	4194	19.36%	18.84 – 19.89%	613121	64.56%	64.46 – 64.65%	3453741	49.01%	48.97 – 49.04%
	Total		5977652	84.82%		98161	1.39%	1.38– 1.40%	21660	0.31%	0.30 – 0.31%	949765	13.48%		7047238	100%	
Ethnicity		White	4438545	74.25%	74.22 – 74.29%	76272	77.7%	77.44 – 77.96%	16003	73.88%	73.29 – 74.46%	721454	75.96%	75.88 – 76.05%	5252274	74.53%	74.50 – 74.56%
		Asian	660235	11.05%	11.02– 11.07%	6416	6.54%	6.38 – 6.69%	1871	8.64%	8.27 – 9.02%	83184	8.76%	8.70 – 8.82%	751706	10.67%	10.64 – 10.69%
		Black	333120	5.57%	5.55 – 5.59%	6647	6.77%	6.61 – 6.93%	1818	8.39%	8.03 – 8.77%	57072	6.01%	5.96 – 6.06%	398657	5.66%	5.64 – 5.67%
		Chinese	27261	0.46%	0.45 – 0.46%	405	0.41%	3.74 – 4.55%	95	0.44%	0.36 – 0.54%	2067	0.22%	0.21 – 0.23%	29828	0.42%	0.42 – 0.43%
		Roma/Irish Traveller	19265	0.32%	0.32 – 0.33%	147	0.15%	0.13 – 0.18%	51	0.24%	0.18 – 0.31%	8149	0.86%	0.84 – 0.88%	27612	0.39%	0.39 – 0.40%
		Mixed	327212	5.47%	5.46 – 5.49%	5818	5.93%	5.78 – 6.08%	1237	5.71%	5.41 – 6.03%	52428	5.52%	5.47 – 5.56%	386695	5.49%	5.47 – 5.50%
		Other	109084	1.82%	1.81 – 1.84%	1272	1.3%	1.23 – 1.37%	327	1.51%	1.36 – 1.68%	15595	1.64%	1.62 – 1.67%	126278	1.79%	1.78 – 1.80%
		Unclassified	62930	1.05%	1.04 – 1.06%	1184	1.21%	1.14 – 1.28%	258	1.19%	1.05 – 1.34%	9816	1.03%	1.01 – 1.05%	74188	1.05%	1.05 – 1.06%
Age	5–9		2728222	45.64%	45.60 – 45.68%	41309	42.08%	41.77 – 42.39%	6757	31.2%	30.58 – 31.82%	452147	47.61%	47.51 – 47.71%	3228435	45.81%	45.77 – 45.85%
	10–14		2394755	40.06%	40.02 – 40.10%	43595	44.41%	44.10 – 44.72%	9750	45.01%	44.35 – 45.68%	395808	41.67%	41.58 – 41.77%	2843908	40.35%	40.32 – 40.39%
	15–19		854675	14.3%	14.27 – 14.33%	13257	13.51%	13.29 – 13.72%	5153	23.79%	23.23 – 24.36%	101810	10.72%	10.66 – 10.78%	974895	13.83%	13.81 – 13.86%
Primary Language	English																
		White	4119977	85.22%	85.19 – 85.25%	73489	85.04%	84.80 – 85.28%	15457	83.04%	82.49 – 83.57%	680518	86.01%	85.94 – 86.09%	4889441	85.32%	85.29 – 85.35%
		Asian	175326	3.63%	3.61 – 3.64%	2061	2.39%	2.29 – 2.49%	579	3.11%	2.87 – 3.37%	17120	2.16%	2.13 – 2.20%	195086	3.4%	3.39 – 3.42%
		Black	184815	3.82%	3.81 – 3.84%	4150	4.8%	4.66 – 4.94%	1109	5.96%	5.63 – 6.31%	32585	4.12%	4.08 – 4.16%	222659	3.89%	3.87 – 3.90%
		Chinese	6602	0.14%	0.13 – 0.14%	105	0.12%	0.10 – 0.15%	20	0.11%	0.07 – 0.17%	341	0.04%	0.04 – 0.05%	7068	0.12%	0.12 – 0.13%
		Roma/Irish Traveller	9874	0.2%	0.20 – 0.21%	126	0.15%	0.12 – 0.17%	44	0.24%	0.18 – 0.32%	5193	0.66%	0.64 – 0.67%	15237	0.27%	0.26 – 0.27%
		Mixed	269250	5.57%	5.55 – 5.59%	5107	5.91%	5.75 – 6.07%	1081	5.81%	5.48 – 6.15%	44966	5.68%	5.63 – 5.73%	320404	5.59%	5.57 – 5.61%
		Other	21053	0.44%	0.43 – 0.44%	388	0.45%	0.41 – 0.50%	112	0.6%	0.50 – 0.72%	2602	0.33%	0.32 – 0.34%	24155	0.42%	0.42 – 0.43%
		Unclassified	47501	0.98%	0.97 – 0.99%	987	1.14%	1.07 – 1.21%	213	1.14%	1.00 – 1.31%	7842	0.99%	0.97 – 1.01%	56543	0.99%	0.98 – 0.99%
		Proportion from Total	4834398	68.6%		86413	1.23%		18615	0.26%		791167	11.23%		5730593	81.32%	81.29 – 81.35%
	Other																
		White	316381	27.93%	27.84 – 28.01%	2745	23.72%	22.95 – 24.50%	543	17.94%	16.62 – 19.35%	40601	25.83%	25.61 – 26.04%	360270	27.61%	27.54 – 27.69%
		Asian	483043	42.64%	42.55 – 42.73%	4333	37.44%	36.56 – 38.33%	1285	42.47%	40.71 – 44.24%	65839	41.88%	41.64 – 42.12%	554500	42.50%	42.42 – 42.59%
		Black	146694	12.95%	12.89 – 13.01%	2461	21.27%	20.53 – 22.02%	708	23.4%	21.92 – 24.94%	24262	15.43%	15.26 – 15.61%	174125	13.35%	13.29 – 13.40%
		Chinese	20549	1.81%	1.79 – 1.84%	299	2.58%	2.31 – 2.89%	75	2.48%	1.98 – 3.10%	1713	1.09%	1.04 – 1.14%	22636	1.73%	1.71 – 1.76%
		Roma/Irish Traveller	9355	0.83%	0.81 – 0.84	21	0.18%	0.12 – 0.28%	12	0.28%	0.11 – 0.48%	2946	1.87%	1.81 – 1.94%	12329	0.94%	0.93 – 0.96%
		Mixed	57308	5.06%	5.02 – 5.1%	696	6.01%	5.60 – 6.46%	156	5.16%	4.42 – 6.00%	7365	4.68%	4.58 – 4.79%	65525	5.02%	4.98 – 5.06%
		Other	87585	7.73%	7.68 – 7.78%	882	7.62%	7.15 – 8.12%	215	7.11%	6.24 – 8.08%	12932	8.23%	8.09 – 8.36%	101614	7.79%	7.74 – 7.83%
		Unclassified	11959	1.06%	1.04 – 1.07%	136	1.18%	0.99 – 1.39%	37	1.22%	0.89 – 1.68%	1548	0.98%	0.94 – 1.03%	13680	1.05%	1.03 – 1.07%
		Proportion from Total	1132874	16.08%		11573	0.16%		3026	0.04%		157206	2.23%		1304679	18.51%	18.48 – 18.54%
	Unknown (% From total School Children)		10380	0.15%		175	0.002%		19	0.0003%		1392	0.02%		11966	0.17%	0.17 – 0.17%
SEND Type	Autism with a Second SEND Total		..	..	..	64212	65.41%	65.11 – 65.71%	..	..	..	..	..	..	119821	1.7%	1.70 – 1.71%
	Sensory Impairment		..	..	..	1154	1.18%	1.11 – 1.25%	..	..	..	42642	4.49%	4.48 – 4.53%	43796	0.62%	0.62 – 0.63%
		Hearing Impairment	..	..	..	458	0.47%	0.43 – 0.51%	..	..	..	23825	2.51%	2.48 – 2.54%	24283	0.34%	0.34 – 0.35%
		Visual Impairment	..	..	..	453	0.46%	0.42 – 0.51%	..	..	..	15556	1.64%	1.61 – 1.66%	16009	0.23%	0.22 – 0.23%
		Multisensory Impairment	..	..	..	243	0.25%	0.22 – 0.28%	..	..	..	3794	0.4%	0.39 – 0.41%	4037	0.057%	0.056 – 0.059%
	Learning Disability		..	..	..	..	..	..	21660	100,00%		484072	50.97%	50.87 – 51.07%	505732	7.18%	7.15 – 7.20%
		Specific	..	..	..	..	..	..	3551	16.39%	15.91 – 16.89%	163407	17.2%	17.13 – 17.28%	166958	2.37%	2.36 – 2.38%
		Moderate	..	..	..	..	..	..	7764	35.84%	35.21 – 36.47%	291189	30.66%	30.57 – 30.75%	298953	4.24%	4.23 – 4.26%
		Severe	..	..	..	..	..	..	9642	44.52%	43.85 – 45.18%	27688	2.92%	2.88 – 2.95%	37330	0.53%	0.52 – 0.54%
		Profound and Multiple	..	..	..	..	..	..	703	3.25%	3.01 – 3.49%	9834	1.04%	1.02 – 1.06%	10537	0.15%	0.15 – 0.15%
	Speech. Language, and Communication		..	..	..	15851	16.15%	15.92 – 16.38%					24.46%		248139		3.51 – 3.53%
	Needs								..	..	..	232288		24.37 – 24.54%		3.52%	
	Physical Disability		..	..	..	1216	1.24%	1.17 – 1.31%	..	..	..	40779	4.29%	4.25 – 4.33%	41995	0.6%	0.59 – 0.60%
	Other		..	..	..	3081	3.14%	3.03 – 3.25%	..	..	..	66734	7.03%	6.98 – 7.08%	69815	0.99%	0.98 – 1.00%
	Social, Emotional, and Mental Health		..	..	..	12442	12.68%	12.47 – 12.88%	..	..	..	223267	23.51%	23.42 – 23.59%	235709	3.34%	3.33 – 3.35%
	No Specialist Assessment		..	..	..	205	0.21%	0.18 – 0.24%	..	..	..	43238	4.55%	4.51 – 4.59%	43443	0.62%	0.61 – 0.62%
EHCP																	
		White	..	..	..	38090	72.76%	72.37 – 73.13%	12291	71.12%	70.44 – 71.79%	109972	75.76%	75.54 – 75.98%	160359	2.28%	2.26 – 2.29%
		Asian	..	..	..	4252	8.12%	7.89 – 8.36%	1669	9.66%	9.22 – 10.11%	14128	9.73%	9.58 – 9.89%	20050	0.28%	0.28 – 0.29%
		Black	..	..	..	4748	9.07%	8.82 – 9.32%	1676	9.7%	9.27 – 10.15%	8138	5.61%	5.49 – 5.73%	14562	0.21%	0.20 – 0.21%
		Chinese	..	..	..	274	0.52%	0.47 – 0.59%	88	0.51%	0.41 – 0.63%	346	0.24%	0.21 – 0.26%	708	0.01%	0.01 – 0.01%
		Roma/Irish Traveller	..	..	..	105	0.2%	0.17 – 0.24%	38	0.22%	0.16 – 0.30%	847	0.58%	0.55 – 0.62%	990	0.01%	0.01 – 0.01%
		Mixed	..	..	..	3314	6.33%	6.12 – 6.54%	1034	5.98%	5.64 – 6.35%	7764	5.35%	5.23 – 5.47%	12112	0.17%	0.17 – 0.17%
		Other	..	..	..	851	1.63%	1.52 – 1.74%	289	1.67%	1.49 – 1.87%	2182	1.5%	1.44 – 1.57%	3324	0.05%	0.05 – 0.05%
		Unclassified	..	..	..	719	1.37%	1.28 – 1.48%	198	1.15%	1.00 – 1.32%	1778	1.22%	1.17 – 1.29%	2696	0.04%	0.04 – 0.04%
SEND Support																	
		White	..	..	..	38182	83.35%	83.01 – 83.69%	3712	84.81%	83.71 – 85.84%	611482	76,00%	75.90 – 76.09%	653376	9.27%	9.25 – 9.29%
		Asian	..	..	..	2164	4.72%	4.53 – 4.92%	202	4.62%	4.03 – 5.28%	69056	8.58%	8.52 – 8.64%	71422	1.01%	1.01 – 1.02%
		Black	..	..	..	1899	4.15%	3.97 – 4.33%	142	3.24%	2.76 – 3.81%	48934	6.08%	6.03 – 6.13%	50975	0.72%	0.72 – 0.73%
		Chinese	..	..	..	131	0.29%	0.24 – 0.34%	133	0.21%	0.08 – 0.34%	1721	0.21%	0.20 – 0.22%	1859	0.03%	0.03 – 0.03%
		Roma/Irish Traveller	..	..	..	42	0.09%	0.07 – 0.12%	13	0.3%	0.17 – 0.51%	7302	0.91%	0.89 – 0.93%	7357	0.1%	0.10 – 0.11%
		Mixed	..	..	..	2504	5.47%	5.26 – 5.68%	203	4.64%	4.05 – 5.30%	44664	5.55%	5.50 – 5.60%	47371	0.67%	0.67 – 0.68%
		Other	..	..	..	421	0.92%	0.84 – 1.01%	38	0.87%	0.63 – 1.19%	13413	1.67%	1.64 – 1.70%	13872	0.2%	0.19 – 0.20%
		Unclassified	..	..	..	465	1.02%	0.93 – 1.11%	60	1.37%	1.07 – 1.76%	8038	1,00%	0.98 – 1.02%	8563	0.12%	0.12 – 0.12%
Claimed Eligibility for Free School Meal	Yes		1365303	100,00%		34587			8215			410082			1818195		
		White	898399	65.8%	65.72 – 65.88%	25490	73.7%	73.23 – 74.16%	5859	71.32%	70.33 – 72.29%	302168	73.68%	73.55 – 73.82%	1231921	67.76%	67.69 – 67.82%
		Asian	151441	11.09%	11.04 – 11.14%	1784	5.16%	4.93 – 5.40%	544	6.62%	6.10 – 7.18%	28887	7.04%	6.97 – 7.12%	182657	10.05%	10.00 – 10.09%
		Black	142696	10.45%	10.40 – 10.50%	3550	10.26%	9.95 – 10.59%	929	11.31%	10.64 – 12.01%	32485	7.92%	7.84 – 8.00%	179660	9.88%	9.84 – 9.92%
		Chinese	3513	0.26%	0.25 – 0.27%	95	0.27%	0.22 – 0.34%	25	0.3%	0.21 – 0.45%	396	0.1%	0.09 – 0.11%	4029	0.22%	0.21 – 0.23%
		Roma/Irish Traveller	9264	0.68%	0.66 – 0.69%	86	0.25%	0.20 – 0.31%	31	0.38%	0.27 – 0.54%	5417	1.32%	1.29 – 1.37%	14798	0.81%	0.80 – 0.83%
		Mixed	105382	7.72%	7.67 – 7.76%	2592	7.49%	7.22 – 7.78%	573	6.98%	6.44 – 7.55%	28562	6.96%	6.89 – 7.04%	137109	7.54%	7.50 – 7.58%
		Other	37912	2.78%	2.75 – 2.80%	501	1.45%	1.32 – 1.58%	141	1.72%	1.46 – 2.02%	7548	1.84%	1.80 – 1.88%	46103	2.54%	2.51 – 2.56%
		Unclassified	16696	1.22%	1.20 – 1.24%	489	1.41%	1.29 – 1.54%	113	1.38%	1.15 – 1.65%	4619	1.13%	1.09 – 1.16%	21918	1.21%	1.19 – 1.22%
	No																
		White	3253306	77.18%	77.14 – 77.22%	46293	79.75%	79.41 – 80.07%	7924	75.55%	74.71 – 76.36%	396418	77.84%	77.73 – 77.96%	3703941	77.28%	77.24 – 77.31%
		Asian	458619	10.88%	10.85 – 10.91%	4353	7.5%	7.29 – 7.72%	1073	10.23%	9.66 – 10.82%	51148	10.04%	9.96 – 10.13%	515193	10.75%	10.72 – 10.78%
		Black	166154	3.94%	3.92 – 3.96%	2792	4.81%	4.64 – 4.99%	626	5.97%	5.53 – 6.43%	22765	4.47%	4.41 – 4.53%	192337	4.01%	4.00 – 4.03%
		Chinese	21092	0.5%	0.49 – 0.51%	290	0.5%	0.45 – 0.56%	58	0.55%	0.43 – 0.71%	1559	0.31%	0.29 – 0.32%	22999	0.48%	0.47 – 0.49%
		Roma/Irish Traveller	9827	0.23%	0.23 – 0.24%	56	0.1%	0.07 – 0.13%	16	0.15%	0.09 – 0.25%	2667	0.52%	0.50 – 0.54%	12566	0.26%	0.26 – 0.27%
		Mixed	202728	4.81%	4.79 – 4.83%	2947	5.08%	4.90 – 5.23%	524	5,00%	4.59 – 5.43%	22445	4.41%	4.35 – 4.46%	228644	4.77%	4.75 – 4.79%
		Other	63345	1.5%	1.49 – 1.51%	709	1.22%	1.14 – 1.31%	148	1.41%	1.20 – 1.66%	7432	1.46%	1.43 – 1.49%	71634	1.49%	1.48 – 1.51%
		Unclassified	40196	0.95%	0.94 – 0.96%	609	1.05%	0.97 – 1.14%	120	1.14%	0.96 – 1.37%	4827	0.95%	0.92 – 0.97%	45752	0.95%	0.95 – 0.96%
	Missing (% From total)		397082	5.63%		5525	0.08%		2956	0.04%		30422	0.43%		435977	6.19%	

©2021 Roman-Urrestarazu A et al. *JAMA Pediatrics*.

Table 2. Sensitivity Analysis for Adjusted Prevalence Ratios Log-Binomial and Poisson Regression with Robust Error Variance in Missing Information and Complete Case Analysis Model for Autism Status as Outcome.

Autism Status		**1. Log-binomial regression FSM With No Missing Cases (Missing information assumed no Free school meals)**<br>**Liberal Assumption**		**2. Poisson regression with robust error variance model, FSM With No Missing Cases (Missing information assumed no Free school meals)**<br>**Liberal Assumption**		**3. Log-binomial regression FSM With Missing Cases (Complete case analysis)**<br>**Conservative Assumption**		**4. Poisson regression with robust error variance model, FSM With Missing Cases (Complete case analysis)**<br>**Conservative Assumption**	
N (%)		7 047 238 (100%)		7 047 238 (100%)		6 611 261 (93.81%)		6 611 261 (93.81%)	
		APR	95% Confidence Interval	APR	95% Confidence Interval	APR	95% Confidence Interval	APR	95% Confidence Interval
Age	5–9	R		R		R		R	
	10–14	1.19 *	1.17 – 1.20	1.19 *	1.18 – 1.21	1.19 *	1.18 – 1.21	1.19 *	1.18 – 1.21
	15–19	1.06 *	1.04 – 1.08	1.06 *	1.04 – 1.08	1.17 *	1.14 – 1.19	1.17 *	1.14 – 1.19
Gender	Female	R		R		R		R	
	Male	4.40 *	4.33 – 4.46	4.40 *	4.33 – 4.46	4.39 *	4.33 – 4.46	4.39 *	4.33 – 4.46
Ethnicity	White	R		R		R		R	
	Asian	0.82 *	0.80 – 0.85	0.83 *	0.81 – 0.85	0.85 *	0.83 – 0.87	0.85 *	0.83 – 0.87
	Black	1.27 *	1.25 – 1.30	1.28 *	1.25 – 1.31	1.28 *	1.25 – 1.31	1.28 *	1.25 – 1.31
	Chinese	1.35 *	1.24 – 1.47	1.35 *	1.24 – 1.48	1.46 *	1.33 – 1.59	1.46 *	1.33 – 1.59
	Roma/Irish Traveller	0.42 *	0.37 – 0.49	0.42 *	0.37 – 0.49	0.40 *	0.35 – 0.47	0.40 *	0.35 – 0.47
	Mixed	1.03	1.01 – 1.05	1.03	1.01 – 1.06	1.03	1.01 – 1.06	1.03	1.01 – 1.06
	Other	0.92	0.88 – 0.97	0.92 *	0.88 – 0.97	0.94	0.89 – 0.99	0.93	0.89 – 0.99
	Unclassified	1.09	1.03 – 1.15	1.09	1.04 – 1.15	1.11 *	1.05 – 1.17	1.11 *	1.05 – 1.17
Claimed Eligibility for Free School Meals	No	R		R		R		R	
	Yes	1.58 *	1.56 – 1.60	1.58 *	1.56 – 1.60	1.61 *	1.59 – 1.63	1.61 *	1.59 – 1.63
Language at Home	English	R		R		R		R	
	Other	0.64 *	0.63 – 0.65	0.64 *	0.63 – 0.65	0.64 *	0.63 – 0.65	0.64 *	0.63 – 0.65
	Unclassified	0.76 *	0.66 – 0.88	0.76 *	0.66 – 0.88	0.86	0.74 – 0.99	0.86	0.74 – 1.00

* $p < 0.001$

©2021 Roman-Urrestarazu A et al. *JAMA Pediatrics*.

Table 3. Sensitivity Analysis for Adjusted Prevalence Ratios Poisson Regression with MICE Estimation Using Autism Status as Outcome and Including Local Authority Districts Decile Index of Multiple Deprivation.

**Autism Status**		**Poisson Regression with MICE Estimation**	
**N (%)**		7,047,238 (100%)	
		APR	95% Confidence Interval
**Age**	5–9	R	
	10–14	1.21*	1.18 – 1.23
	15–19	1.08*	1.04 – 1.12
**Gender**	Female	R	
	Male	3.76*	3.68 – 3.85
**Ethnicity**	White	R	
	Asian	0.58*	0.55 – 0.60
	Black	0.71*	0.68 – 0.74
	Chinese	0.98	0.83 – 1.17
	Roma/Irish Traveller	0.27*	0.20 – 0.35
	Mixed	0.87*	0.84 – 0.90
	Other	0.66*	0.59 – 0.72
	Unclassified	0.89	0.82 – 0.98
**Deciles of Index of Multiple Deprivation**	10 (wealthiest)	R	1.21 - 1.32
	9	1.02*	0.98 - 1.07
	8	1.09*	1.04 - 1.14
	7	1.14*	1.09 - 1.19
	6	1.18*	1.13 - 1.23
	5	1.20*	1.15 - 1.25
	4	1.19*	1.14 - 1.24
	3	1.25*	1.20 - 1.31
	2	1.29*	1.23 - 1.34
	1 (poorest)	1.26*	1.21 - 1.32
**Claimed Eligibility for Free School Meals**	No	R	
	Yes	1.40*	1.37 – 1.43
**Language at Home**	English	R	
	Other	0.53*	0.51 – 0.55
	Unclassified	1.03	0.83 – 1.29

* $p < 0.001$

Table 4. Age and Sex Standardised Prevalence of ASD by Local Authority District

LAD	Total Population				Female				Male				M2 FR
	Crude Prevalence	Adjusted Prevalence	Confidence Interval		Crude Prevalence	Adjusted Prevalence	Confidence Interval		Crude Prevalence	Adjusted Prevalence	Confidence Interval		
Adur	1.27%	1.42%	1.10%	1.75%	1.98%	1.12%	0.83%	1.41%	0.51%	0.30%	0.15%	0.45%	3.76
Allerdale	1.63%	1.65%	1.40%	1.91%	2.72%	1.40%	1.17%	1.64%	0.49%	0.25%	0.15%	0.35%	5.62
Amber Valley	2.04%	2.19%	1.92%	2.45%	3.24%	1.77%	1.53%	2.01%	0.80%	0.42%	0.30%	0.54%	4.20
Arun	1.24%	1.33%	1.13%	1.53%	2.04%	1.14%	0.95%	1.33%	0.38%	0.19%	0.12%	0.27%	5.91
Ashfield	2.58%	2.74%	2.44%	3.03%	4.16%	2.25%	1.98%	2.51%	0.95%	0.49%	0.37%	0.61%	4.61
Ashford	1.85%	1.87%	1.66%	2.08%	3.07%	1.58%	1.39%	1.77%	0.60%	0.29%	0.21%	0.37%	5.44
Aylesbury Vale	1.60%	1.70%	1.53%	1.87%	2.52%	1.37%	1.22%	1.52%	0.63%	0.33%	0.25%	0.41%	4.14
Babergh	1.88%	1.86%	1.59%	2.14%	3.09%	1.54%	1.29%	1.78%	0.62%	0.33%	0.20%	0.45%	4.68
Barking and Dagenham	1.68%	1.53%	1.40%	1.66%	2.81%	1.31%	1.19%	1.43%	0.50%	0.22%	0.17%	0.27%	6.02
Barnet	1.53%	1.53%	1.41%	1.65%	2.50%	1.28%	1.17%	1.39%	0.51%	0.25%	0.20%	0.30%	5.12
Barnsley	1.74%	1.89%	1.69%	2.09%	2.84%	1.58%	1.39%	1.76%	0.59%	0.31%	0.23%	0.39%	5.07
Barrow-in-Furness	1.70%	2.12%	1.70%	2.54%	2.77%	1.68%	1.31%	2.04%	0.55%	0.44%	0.23%	0.66%	3.77
Basildon	1.94%	1.91%	1.73%	2.10%	2.99%	1.52%	1.35%	1.69%	0.85%	0.39%	0.31%	0.47%	3.89
Basingstoke and Deane	1.65%	1.89%	1.64%	2.15%	2.67%	1.56%	1.34%	1.79%	0.57%	0.33%	0.22%	0.44%	4.73
Bassetlaw	1.55%	1.59%	1.36%	1.82%	2.45%	1.28%	1.07%	1.49%	0.63%	0.31%	0.21%	0.41%	4.17
Bath and North East Somerset	1.91%	1.97%	1.76%	2.18%	2.99%	1.56%	1.38%	1.75%	0.77%	0.41%	0.31%	0.50%	3.84
Bedford	1.92%	1.91%	1.72%	2.10%	3.22%	1.65%	1.47%	1.83%	0.59%	0.26%	0.19%	0.33%	6.31
Bexley	2.38%	2.40%	2.23%	2.57%	3.77%	1.90%	1.75%	2.05%	0.96%	0.50%	0.42%	0.57%	3.84
Birmingham	2.27%	2.44%	2.35%	2.52%	3.58%	1.97%	1.89%	2.05%	0.91%	0.47%	0.43%	0.51%	4.20
Blaby	1.35%	1.38%	1.15%	1.61%	2.16%	1.12%	0.91%	1.32%	0.52%	0.26%	0.16%	0.36%	4.27
Blackburn with Darwen	0.94%	1.08%	0.91%	1.25%	1.62%	0.93%	0.77%	1.09%	0.24%	0.15%	0.08%	0.22%	6.21
Blackpool	1.07%	1.31%	1.07%	1.55%	1.74%	1.13%	0.90%	1.36%	0.37%	0.18%	0.10%	0.26%	6.26
Bolsover	1.64%	1.70%	1.40%	2.01%	2.75%	1.42%	1.14%	1.70%	0.52%	0.28%	0.15%	0.41%	5.05
Bolton	1.21%	1.33%	1.19%	1.47%	1.96%	1.11%	0.98%	1.24%	0.41%	0.22%	0.16%	0.28%	5.00
Boston	1.28%	1.30%	1.04%	1.57%	2.08%	1.12%	0.87%	1.37%	0.44%	0.19%	0.10%	0.27%	5.96
Bournemouth	1.70%	1.75%	1.54%	1.95%	2.72%	1.40%	1.22%	1.58%	0.64%	0.35%	0.25%	0.44%	4.01
Bracknell Forest	2.43%	2.62%	2.33%	2.91%	3.78%	2.05%	1.80%	2.31%	1.04%	0.57%	0.43%	0.70%	3.63
Bradford	1.24%	1.29%	1.21%	1.38%	2.02%	1.06%	0.98%	1.14%	0.44%	0.23%	0.19%	0.27%	4.62
Braintree	2.16%	2.17%	1.93%	2.40%	3.48%	1.77%	1.56%	1.98%	0.80%	0.39%	0.29%	0.49%	4.50
Breckland	1.54%	1.53%	1.32%	1.74%	2.60%	1.32%	1.12%	1.52%	0.44%	0.21%	0.13%	0.28%	6.42
Brent	1.47%	1.41%	1.29%	1.52%	2.46%	1.21%	1.10%	1.32%	0.44%	0.20%	0.16%	0.24%	6.03
Brentwood	2.18%	2.14%	1.83%	2.44%	3.70%	1.84%	1.56%	2.13%	0.64%	0.29%	0.18%	0.40%	6.29
Brighton and Hove	1.45%	1.55%	1.37%	1.73%	2.36%	1.32%	1.16%	1.49%	0.49%	0.23%	0.16%	0.30%	5.76
Bristol, City of	1.76%	1.75%	1.62%	1.88%	2.75%	1.41%	1.29%	1.53%	0.74%	0.33%	0.28%	0.39%	4.23
Broadland	1.04%	1.03%	0.85%	1.21%	1.63%	0.84%	0.68%	1.00%	0.41%	0.19%	0.12%	0.27%	4.34
Bromley	2.02%	2.07%	1.92%	2.21%	3.22%	1.68%	1.55%	1.81%	0.79%	0.39%	0.33%	0.45%	4.34
Bromsgrove	1.88%	1.98%	1.70%	2.26%	2.94%	1.59%	1.34%	1.84%	0.74%	0.39%	0.26%	0.51%	4.12
Broxbourne	1.37%	1.32%	1.12%	1.53%	2.27%	1.15%	0.96%	1.34%	0.43%	0.18%	0.11%	0.24%	6.47
Broxtowe	1.55%	1.63%	1.38%	1.89%	2.45%	1.31%	1.09%	1.54%	0.60%	0.32%	0.21%	0.44%	4.10
Burnley	1.24%	1.24%	1.01%	1.48%	2.18%	1.16%	0.93%	1.39%	0.23%	0.09%	0.04%	0.13%	13.57
Bury	1.18%	1.29%	1.11%	1.48%	1.89%	1.04%	0.88%	1.21%	0.44%	0.25%	0.17%	0.34%	4.11
Calderdale	0.95%	1.00%	0.87%	1.13%	1.60%	0.85%	0.74%	0.97%	0.30%	0.14%	0.10%	0.19%	5.93
Cambridge	1.63%	1.84%	1.51%	2.18%	2.65%	1.55%	1.24%	1.85%	0.55%	0.30%	0.16%	0.43%	5.25
Camden	1.57%	1.59%	1.39%	1.79%	2.54%	1.30%	1.12%	1.48%	0.58%	0.29%	0.21%	0.37%	4.49
Cannock Chase	2.31%	2.40%	2.09%	2.71%	3.71%	1.97%	1.68%	2.25%	0.85%	0.44%	0.31%	0.57%	4.51
Canterbury	2.20%	2.22%	1.98%	2.45%	3.38%	1.75%	1.54%	1.95%	0.94%	0.47%	0.36%	0.57%	3.74
Carlisle	0.83%	0.86%	0.68%	1.04%	1.35%	0.69%	0.53%	0.86%	0.30%	0.17%	0.09%	0.25%	4.18
Castle Point	1.08%	1.23%	0.98%	1.49%	1.74%	0.99%	0.77%	1.22%	0.40%	0.24%	0.13%	0.35%	4.12
Central Bedfordshire	2.23%	2.26%	2.09%	2.43%	3.56%	1.83%	1.68%	1.98%	0.84%	0.43%	0.35%	0.50%	4.28
Charnwood	1.49%	1.70%	1.48%	1.92%	2.36%	1.39%	1.19%	1.59%	0.59%	0.31%	0.22%	0.40%	4.48
Chelmsford	1.57%	1.51%	1.35%	1.68%	2.52%	1.25%	1.10%	1.40%	0.56%	0.26%	0.19%	0.33%	4.79
Cheltenham	0.87%	0.87%	0.70%	1.05%	1.44%	0.75%	0.59%	0.92%	0.28%	0.12%	0.06%	0.18%	6.32
Cherwell	1.43%	1.51%	1.31%	1.71%	2.29%	1.26%	1.08%	1.45%	0.54%	0.25%	0.18%	0.32%	5.06
Cheshire East	1.18%	1.18%	1.07%	1.29%	1.91%	0.97%	0.87%	1.07%	0.41%	0.21%	0.16%	0.26%	4.65
Cheshire West and Chester	1.61%	1.66%	1.52%	1.80%	2.55%	1.34%	1.22%	1.47%	0.61%	0.32%	0.25%	0.38%	4.26
Chesterfield	1.12%	1.08%	0.88%	1.27%	1.97%	0.95%	0.77%	1.14%	0.26%	0.12%	0.06%	0.19%	7.84
Chichester	1.16%	1.25%	1.01%	1.49%	1.82%	1.01%	0.79%	1.23%	0.47%	0.24%	0.13%	0.34%	4.26
Chiltern	1.19%	1.23%	1.03%	1.43%	1.82%	0.93%	0.76%	1.11%	0.53%	0.30%	0.19%	0.40%	3.15
Chorley	1.57%	1.93%	1.60%	2.27%	2.63%	1.71%	1.39%	2.02%	0.44%	0.22%	0.12%	0.33%	7.62
Christchurch	1.10%	1.13%	0.82%	1.43%	1.71%	0.87%	0.61%	1.14%	0.44%	0.25%	0.10%	0.41%	3.44
City of London	1.88%	1.73%	0.22%	3.24%	2.82%	1.26%	0.05%	2.47%	0.81%	0.47%	0.00%	1.37%	2.71
Colchester	2.09%	2.02%	1.81%	2.24%	3.34%	1.65%	1.46%	1.85%	0.77%	0.37%	0.28%	0.47%	4.44
Copeland	2.30%	2.28%	1.93%	2.63%	3.62%	1.85%	1.53%	2.17%	0.91%	0.43%	0.28%	0.58%	4.31
Corby	1.23%	1.20%	0.96%	1.44%	2.07%	1.06%	0.83%	1.29%	0.37%	0.14%	0.08%	0.20%	7.56
Cornwall	1.34%	1.39%	1.28%	1.50%	2.16%	1.14%	1.05%	1.24%	0.49%	0.25%	0.20%	0.29%	4.66
Cotswold	0.63%	0.63%	0.46%	0.81%	1.05%	0.54%	0.37%	0.70%	0.21%	0.09%	0.03%	0.16%	5.73
County Durham	1.60%	1.66%	1.55%	1.78%	2.58%	1.37%	1.26%	1.47%	0.58%	0.29%	0.25%	0.34%	4.66
Coventry	2.98%	3.08%	2.90%	3.25%	4.65%	2.47%	2.31%	2.63%	1.25%	0.61%	0.53%	0.69%	4.05
Craven	1.75%	1.77%	1.42%	2.11%	2.42%	1.28%	0.98%	1.57%	1.02%	0.49%	0.31%	0.67%	2.58
Crawley	1.38%	1.36%	1.17%	1.55%	2.19%	1.11%	0.94%	1.29%	0.53%	0.25%	0.16%	0.33%	4.55
Croydon	2.00%	2.02%	1.90%	2.15%	3.33%	1.70%	1.58%	1.82%	0.65%	0.32%	0.27%	0.38%	5.23
Dacorum	1.65%	1.62%	1.43%	1.81%	2.73%	1.37%	1.20%	1.55%	0.55%	0.25%	0.18%	0.32%	5.45
Darlington	1.62%	1.83%	1.52%	2.14%	2.68%	1.49%	1.21%	1.76%	0.51%	0.34%	0.20%	0.49%	4.32
Dartford	2.78%	2.82%	2.53%	3.11%	4.50%	2.33%	2.07%	2.60%	1.00%	0.48%	0.36%	0.60%	4.82
Daventry	1.47%	1.51%	1.25%	1.76%	2.23%	1.17%	0.94%	1.39%	0.68%	0.34%	0.22%	0.46%	3.45
Derby	1.97%	2.08%	1.91%	2.26%	3.14%	1.72%	1.56%	1.88%	0.71%	0.36%	0.29%	0.43%	4.77

©2021 Roman-Urrestarazu A et al. *JAMA Pediatrics*.

Derbyshire Dales	1.77%	1.83%	1.52%	2.14%	2.66%	1.42%	1.14%	1.69%	0.85%	0.41%	0.27%	0.56%	3.45
Doncaster	1.89%	1.85%	1.71%	2.00%	3.07%	1.54%	1.41%	1.68%	0.68%	0.31%	0.25%	0.37%	4.93
Dover	1.81%	1.77%	1.54%	1.99%	2.93%	1.47%	1.27%	1.68%	0.66%	0.29%	0.21%	0.38%	5.01
Dudley	1.06%	1.18%	1.05%	1.32%	1.75%	1.02%	0.89%	1.14%	0.34%	0.16%	0.12%	0.21%	6.23
Ealing	1.56%	1.54%	1.42%	1.66%	2.56%	1.30%	1.20%	1.41%	0.50%	0.24%	0.19%	0.28%	5.54
East Cambridgeshire	1.66%	1.93%	1.56%	2.29%	2.66%	1.61%	1.28%	1.95%	0.60%	0.31%	0.18%	0.45%	5.15
East Devon	1.32%	1.29%	1.10%	1.48%	2.00%	1.04%	0.87%	1.21%	0.59%	0.25%	0.18%	0.33%	4.11
East Dorset	0.99%	1.04%	0.83%	1.26%	1.49%	0.80%	0.61%	0.99%	0.47%	0.24%	0.14%	0.35%	3.26
East Hampshire	0.96%	1.11%	0.86%	1.37%	1.65%	0.96%	0.72%	1.19%	0.25%	0.16%	0.06%	0.26%	6.04
East Hertfordshire	1.28%	1.28%	1.12%	1.45%	2.08%	1.05%	0.90%	1.20%	0.46%	0.23%	0.16%	0.31%	4.49
East Lindsey	2.24%	2.33%	2.06%	2.59%	3.39%	1.79%	1.56%	2.02%	1.07%	0.54%	0.41%	0.66%	3.33
East Northamptonshire	1.63%	1.76%	1.48%	2.04%	2.64%	1.41%	1.17%	1.66%	0.59%	0.35%	0.22%	0.48%	4.07
East Riding of Yorkshire	1.07%	1.09%	0.98%	1.21%	1.89%	0.98%	0.87%	1.09%	0.22%	0.11%	0.08%	0.15%	8.57
East Staffordshire	1.34%	1.43%	1.22%	1.64%	2.29%	1.25%	1.05%	1.44%	0.38%	0.19%	0.12%	0.26%	6.72
Eastbourne	2.40%	2.43%	2.07%	2.79%	3.69%	1.86%	1.56%	2.17%	1.00%	0.57%	0.38%	0.75%	3.30
Eastleigh	1.32%	1.37%	1.13%	1.61%	2.27%	1.22%	0.99%	1.44%	0.31%	0.15%	0.07%	0.24%	7.90
Eden	0.80%	0.85%	0.59%	1.11%	1.21%	0.69%	0.45%	0.92%	0.37%	0.16%	0.06%	0.26%	4.19
Elmbridge	2.09%	2.05%	1.79%	2.32%	3.52%	1.76%	1.52%	2.00%	0.59%	0.29%	0.18%	0.39%	6.10
Enfield	1.32%	1.36%	1.25%	1.47%	2.10%	1.11%	1.01%	1.21%	0.50%	0.25%	0.21%	0.30%	4.36
Epping Forest	1.11%	1.06%	0.89%	1.24%	1.81%	0.89%	0.73%	1.05%	0.39%	0.18%	0.11%	0.24%	5.02
Epsom and Ewell	1.49%	1.64%	1.37%	1.91%	2.53%	1.43%	1.18%	1.69%	0.44%	0.20%	0.12%	0.29%	7.03
Erewash	1.78%	1.87%	1.60%	2.14%	2.63%	1.41%	1.18%	1.64%	0.88%	0.46%	0.33%	0.59%	3.07
Exeter	1.55%	1.79%	1.46%	2.12%	2.56%	1.51%	1.21%	1.81%	0.47%	0.28%	0.15%	0.41%	5.46
Fareham	0.79%	0.78%	0.60%	0.97%	1.43%	0.72%	0.54%	0.89%	0.13%	0.07%	0.01%	0.12%	10.93
Fenland	1.67%	1.88%	1.58%	2.17%	2.79%	1.61%	1.34%	1.89%	0.54%	0.26%	0.16%	0.36%	6.12
Forest Heath	1.23%	1.18%	0.88%	1.49%	2.04%	1.00%	0.72%	1.28%	0.41%	0.19%	0.07%	0.30%	5.32
Forest of Dean	0.67%	0.69%	0.50%	0.88%	1.17%	0.61%	0.43%	0.78%	0.17%	0.08%	0.02%	0.14%	7.45
Fylde	1.00%	1.06%	0.78%	1.34%	1.66%	0.84%	0.60%	1.08%	0.34%	0.23%	0.08%	0.37%	3.69
Gateshead	1.66%	1.69%	1.51%	1.88%	2.78%	1.45%	1.28%	1.62%	0.50%	0.24%	0.17%	0.31%	6.06
Gedling	2.34%	2.34%	2.07%	2.61%	3.73%	1.94%	1.69%	2.18%	0.83%	0.40%	0.29%	0.52%	4.80
Gloucester	0.93%	0.90%	0.75%	1.04%	1.56%	0.76%	0.62%	0.89%	0.27%	0.14%	0.08%	0.20%	5.42
Gosport	1.44%	1.30%	1.06%	1.54%	2.34%	1.10%	0.87%	1.32%	0.47%	0.20%	0.11%	0.28%	5.54
Gravesham	3.25%	3.36%	3.05%	3.67%	5.09%	2.67%	2.40%	2.94%	1.32%	0.69%	0.54%	0.83%	3.88
Great Yarmouth	3.00%	3.25%	2.84%	3.67%	4.82%	2.72%	2.34%	3.11%	1.15%	0.53%	0.37%	0.69%	5.14
Greenwich	2.34%	2.31%	2.15%	2.48%	3.77%	1.92%	1.77%	2.07%	0.88%	0.40%	0.33%	0.46%	4.82
Guildford	2.08%	2.08%	1.83%	2.34%	3.26%	1.68%	1.46%	1.91%	0.79%	0.40%	0.28%	0.52%	4.22
Hackney	1.75%	1.76%	1.59%	1.92%	2.96%	1.50%	1.35%	1.65%	0.54%	0.26%	0.20%	0.32%	5.83
Halton	1.52%	1.87%	1.60%	2.13%	2.53%	1.58%	1.34%	1.82%	0.48%	0.29%	0.18%	0.39%	5.54
Hambleton	1.12%	1.20%	0.96%	1.44%	1.83%	1.00%	0.78%	1.22%	0.39%	0.20%	0.10%	0.29%	5.08
Hammersmith and Fulham	1.84%	1.79%	1.57%	2.02%	2.97%	1.48%	1.27%	1.68%	0.68%	0.31%	0.22%	0.41%	4.70
Harborough	1.04%	1.06%	0.86%	1.26%	1.66%	0.86%	0.68%	1.03%	0.37%	0.21%	0.11%	0.30%	4.14
Haringey	1.92%	1.87%	1.71%	2.03%	3.01%	1.49%	1.35%	1.64%	0.81%	0.37%	0.30%	0.44%	4.00
Harlow	1.06%	1.02%	0.82%	1.22%	1.79%	0.88%	0.69%	1.06%	0.30%	0.14%	0.07%	0.22%	6.15
Harrogate	1.73%	1.74%	1.55%	1.94%	2.75%	1.42%	1.24%	1.60%	0.68%	0.32%	0.24%	0.41%	4.40
Harrow	1.66%	1.66%	1.51%	1.82%	2.72%	1.40%	1.26%	1.54%	0.54%	0.26%	0.20%	0.32%	5.32
Hart	1.32%	1.39%	1.13%	1.66%	2.05%	1.17%	0.92%	1.42%	0.54%	0.22%	0.13%	0.31%	5.24
Hartlepool	1.44%	1.51%	1.24%	1.78%	2.37%	1.29%	1.04%	1.55%	0.50%	0.22%	0.13%	0.31%	5.93
Hastings	1.84%	1.89%	1.57%	2.20%	2.92%	1.54%	1.26%	1.82%	0.66%	0.35%	0.21%	0.49%	4.39
Havant	1.23%	1.50%	1.22%	1.78%	1.98%	1.26%	1.00%	1.53%	0.45%	0.23%	0.13%	0.34%	5.40
Havering	1.11%	1.19%	1.05%	1.33%	1.87%	1.01%	0.88%	1.14%	0.35%	0.18%	0.13%	0.23%	5.58
Herefordshire, County of	1.12%	1.37%	1.16%	1.59%	1.76%	1.11%	0.92%	1.31%	0.43%	0.26%	0.17%	0.36%	4.28
Hertsmere	1.57%	1.51%	1.30%	1.71%	2.65%	1.31%	1.12%	1.51%	0.44%	0.19%	0.12%	0.26%	6.84
High Peak	1.40%	1.71%	1.40%	2.03%	2.12%	1.24%	0.98%	1.51%	0.63%	0.47%	0.29%	0.64%	2.66
Hillingdon	2.35%	2.20%	2.05%	2.34%	3.72%	1.78%	1.66%	1.91%	0.91%	0.41%	0.35%	0.47%	4.33
Hinckley and Bosworth	1.27%	1.46%	1.22%	1.70%	2.16%	1.23%	1.01%	1.45%	0.33%	0.23%	0.13%	0.33%	5.37
Horsham	1.33%	1.38%	1.17%	1.58%	2.15%	1.16%	0.97%	1.36%	0.47%	0.21%	0.14%	0.29%	5.51
Hounslow	1.49%	1.50%	1.36%	1.63%	2.51%	1.30%	1.17%	1.43%	0.43%	0.20%	0.15%	0.24%	6.60
Huntingdonshire	1.66%	1.69%	1.51%	1.88%	2.67%	1.41%	1.24%	1.58%	0.59%	0.29%	0.21%	0.36%	4.89
Hyndburn	1.26%	1.38%	1.12%	1.64%	2.04%	1.15%	0.91%	1.40%	0.45%	0.22%	0.13%	0.32%	5.14
Ipswich	1.86%	1.96%	1.73%	2.19%	3.08%	1.65%	1.44%	1.86%	0.58%	0.31%	0.22%	0.40%	5.35
Isle of Wight	1.97%	2.17%	1.90%	2.43%	3.12%	1.76%	1.52%	1.99%	0.75%	0.41%	0.30%	0.53%	4.26
Isles of Scilly	2.75%	3.31%	0.00%	6.90%									
Islington	2.28%	2.31%	2.07%	2.54%	3.75%	1.96%	1.74%	2.17%	0.76%	0.35%	0.26%	0.43%	5.61
Kensington and Chelsea	1.83%	1.83%	1.52%	2.15%	2.92%	1.50%	1.21%	1.78%	0.69%	0.33%	0.20%	0.47%	4.48
Kettering	1.44%	1.57%	1.33%	1.80%	2.35%	1.25%	1.04%	1.47%	0.50%	0.31%	0.20%	0.42%	4.05
King's Lynn and West Norfolk	1.67%	1.77%	1.53%	2.01%	2.76%	1.50%	1.28%	1.72%	0.55%	0.27%	0.19%	0.36%	5.49
Kingston upon Hull, City of	1.65%	1.70%	1.53%	1.87%	2.64%	1.40%	1.25%	1.56%	0.60%	0.30%	0.22%	0.37%	4.75
Kingston upon Thames	2.27%	2.26%	2.04%	2.48%	3.72%	1.90%	1.69%	2.11%	0.81%	0.36%	0.28%	0.45%	5.22
Kirklees	0.63%	0.65%	0.57%	0.73%	0.97%	0.51%	0.44%	0.58%	0.26%	0.14%	0.10%	0.17%	3.77
Knowsley	2.08%	2.22%	1.97%	2.46%	3.37%	1.80%	1.58%	2.02%	0.75%	0.42%	0.31%	0.53%	4.27
Lambeth	2.13%	2.24%	2.06%	2.42%	3.48%	1.87%	1.70%	2.03%	0.77%	0.37%	0.30%	0.44%	5.07
Lancaster	2.23%	2.32%	2.07%	2.57%	3.67%	1.97%	1.74%	2.20%	0.70%	0.35%	0.25%	0.45%	5.63
Leeds	0.86%	0.94%	0.87%	1.01%	1.40%	0.78%	0.72%	0.85%	0.30%	0.15%	0.13%	0.18%	5.05
Leicester	1.07%	1.22%	1.09%	1.35%	1.71%	1.01%	0.90%	1.13%	0.39%	0.21%	0.16%	0.26%	4.90
Lewes	1.73%	1.59%	1.33%	1.85%	2.81%	1.33%	1.10%	1.57%	0.52%	0.26%	0.14%	0.37%	5.22
Lewisham	3.20%	3.19%	2.99%	3.38%	5.12%	2.60%	2.43%	2.78%	1.26%	0.58%	0.50%	0.67%	4.45
Lichfield	1.60%	1.58%	1.35%	1.81%	2.59%	1.30%	1.09%	1.52%	0.57%	0.28%	0.18%	0.37%	4.72
Lincoln	3.05%	3.14%	2.79%	3.50%	4.61%	2.43%	2.12%	2.74%	1.36%	0.72%	0.54%	0.89%	3.39
Liverpool	2.97%	3.00%	2.85%	3.15%	4.64%	2.39%	2.25%	2.52%	1.27%	0.62%	0.55%	0.68%	3.88
Luton	1.48%	1.58%	1.41%	1.75%	2.46%	1.34%	1.19%	1.50%	0.45%	0.23%	0.17%	0.30%	5.74
Maidstone	1.75%	1.92%	1.72%	2.13%	2.77%	1.54%	1.36%	1.73%	0.69%	0.38%	0.29%	0.47%	4.06

©2021 Roman-Urrestarazu A et al. *JAMA Pediatrics*.

Maldon	2.01%	2.01%	1.68%	2.34%	3.17%	1.61%	1.32%	1.90%	0.77%	0.40%	0.25%	0.56%	3.99
Malvern Hills	1.39%	1.55%	1.23%	1.87%	2.11%	1.22%	0.94%	1.51%	0.63%	0.33%	0.18%	0.47%	3.73
Manchester	1.76%	1.84%	1.71%	1.97%	2.90%	1.58%	1.46%	1.70%	0.58%	0.26%	0.22%	0.31%	5.96
Mansfield	2.25%	2.51%	2.19%	2.84%	3.69%	2.07%	1.78%	2.36%	0.80%	0.44%	0.31%	0.58%	4.67
Medway	2.79%	2.99%	2.81%	3.18%	4.36%	2.36%	2.20%	2.53%	1.20%	0.63%	0.55%	0.72%	3.73
Melton	1.33%	1.52%	1.16%	1.89%	2.32%	1.33%	0.99%	1.66%	0.35%	0.20%	0.07%	0.33%	6.74
Mendip	1.38%	1.44%	1.21%	1.67%	2.14%	1.18%	0.97%	1.39%	0.57%	0.26%	0.17%	0.35%	4.48
Merton	1.65%	1.71%	1.53%	1.88%	2.59%	1.39%	1.23%	1.55%	0.67%	0.32%	0.24%	0.39%	4.41
Mid Devon	1.43%	1.38%	1.11%	1.65%	2.35%	1.17%	0.92%	1.42%	0.46%	0.22%	0.11%	0.33%	5.36
Mid Suffolk	1.18%	1.15%	0.95%	1.34%	1.92%	0.93%	0.75%	1.10%	0.43%	0.22%	0.13%	0.31%	4.24
Mid Sussex	1.54%	1.70%	1.47%	1.93%	2.54%	1.43%	1.22%	1.64%	0.48%	0.26%	0.17%	0.36%	5.42
Middlesbrough	1.39%	1.63%	1.41%	1.86%	2.27%	1.38%	1.17%	1.59%	0.49%	0.25%	0.17%	0.33%	5.59
Milton Keynes	1.56%	1.56%	1.44%	1.69%	2.49%	1.29%	1.17%	1.40%	0.57%	0.28%	0.22%	0.33%	4.66
Mole Valley	1.69%	1.74%	1.46%	2.03%	2.91%	1.53%	1.26%	1.79%	0.43%	0.22%	0.12%	0.32%	7.05
New Forest	1.28%	1.32%	1.13%	1.51%	2.03%	1.06%	0.89%	1.23%	0.51%	0.25%	0.17%	0.34%	4.19
Newark and Sherwood	2.30%	2.40%	2.12%	2.68%	3.51%	1.89%	1.64%	2.14%	1.07%	0.51%	0.39%	0.63%	3.69
Newcastle upon Tyne	1.37%	1.43%	1.29%	1.58%	2.25%	1.19%	1.06%	1.32%	0.44%	0.24%	0.18%	0.30%	4.96
Newcastle-under-Lyme	1.20%	1.24%	1.01%	1.46%	1.88%	0.99%	0.79%	1.19%	0.49%	0.24%	0.15%	0.34%	4.05
Newham	1.55%	1.48%	1.36%	1.59%	2.53%	1.21%	1.10%	1.31%	0.55%	0.27%	0.22%	0.32%	4.46
North Devon	1.29%	1.43%	1.14%	1.71%	1.98%	1.15%	0.89%	1.41%	0.58%	0.28%	0.16%	0.39%	4.16
North Dorset	2.11%	2.10%	1.77%	2.44%	3.28%	1.68%	1.38%	1.98%	0.86%	0.42%	0.27%	0.57%	3.99
North East Derbyshire	1.26%	1.35%	1.11%	1.59%	2.08%	1.14%	0.92%	1.37%	0.43%	0.21%	0.12%	0.30%	5.53
North East Lincolnshire	1.23%	1.28%	1.08%	1.48%	1.97%	1.07%	0.88%	1.25%	0.44%	0.21%	0.13%	0.29%	5.06
North Hertfordshire	2.20%	2.23%	1.99%	2.47%	3.55%	1.85%	1.63%	2.07%	0.80%	0.38%	0.28%	0.47%	4.94
North Kesteven	1.76%	1.76%	1.54%	1.98%	2.81%	1.42%	1.22%	1.62%	0.71%	0.34%	0.24%	0.43%	4.23
North Lincolnshire	0.97%	1.09%	0.90%	1.28%	1.60%	0.93%	0.76%	1.10%	0.31%	0.16%	0.09%	0.23%	5.83
North Norfolk	1.54%	1.60%	1.30%	1.90%	2.51%	1.31%	1.05%	1.58%	0.51%	0.29%	0.15%	0.42%	4.60
North Somerset	0.88%	0.95%	0.81%	1.08%	1.51%	0.84%	0.71%	0.97%	0.23%	0.11%	0.07%	0.15%	7.67
North Tyneside	1.28%	1.34%	1.18%	1.50%	2.13%	1.15%	1.01%	1.30%	0.37%	0.19%	0.13%	0.24%	6.21
North Warwickshire	1.76%	1.89%	1.54%	2.25%	2.86%	1.57%	1.24%	1.90%	0.65%	0.32%	0.18%	0.46%	4.92
North West Leicestershire	1.34%	1.47%	1.23%	1.71%	2.02%	1.13%	0.92%	1.35%	0.63%	0.33%	0.22%	0.45%	3.40
Northampton	1.67%	1.71%	1.55%	1.87%	2.65%	1.36%	1.22%	1.50%	0.66%	0.35%	0.28%	0.42%	3.90
Northumberland	1.90%	2.03%	1.87%	2.19%	3.05%	1.69%	1.54%	1.83%	0.70%	0.35%	0.28%	0.41%	4.87
Norwich	1.22%	1.30%	1.08%	1.52%	1.98%	1.08%	0.88%	1.28%	0.44%	0.22%	0.13%	0.31%	4.85
Nottingham	2.42%	2.40%	2.22%	2.58%	3.83%	1.96%	1.79%	2.12%	0.93%	0.45%	0.37%	0.52%	4.39
Nuneaton and Bedworth	2.21%	2.38%	2.10%	2.66%	3.53%	1.96%	1.69%	2.22%	0.90%	0.42%	0.32%	0.53%	4.62
Oadby and Wigston	1.37%	1.40%	1.11%	1.70%	2.44%	1.24%	0.96%	1.51%	0.28%	0.17%	0.06%	0.28%	7.35
Oldham	2.11%	2.24%	2.05%	2.44%	3.40%	1.85%	1.67%	2.02%	0.76%	0.40%	0.31%	0.48%	4.67
Oxford	1.95%	1.99%	1.75%	2.23%	3.05%	1.61%	1.39%	1.82%	0.78%	0.38%	0.28%	0.49%	4.18
Pendle	1.36%	1.48%	1.21%	1.75%	2.18%	1.22%	0.97%	1.47%	0.51%	0.26%	0.15%	0.37%	4.65
Peterborough	1.77%	1.75%	1.59%	1.91%	2.73%	1.39%	1.24%	1.53%	0.76%	0.37%	0.29%	0.44%	3.78
Plymouth	2.45%	2.61%	2.42%	2.80%	3.84%	2.10%	1.93%	2.27%	1.01%	0.51%	0.43%	0.59%	4.10
Poole	1.13%	1.25%	1.06%	1.44%	1.78%	1.01%	0.84%	1.17%	0.45%	0.25%	0.16%	0.33%	4.09
Portsmouth	1.21%	1.25%	1.06%	1.44%	2.01%	1.05%	0.88%	1.23%	0.35%	0.20%	0.11%	0.28%	5.31
Preston	1.47%	1.51%	1.28%	1.74%	2.47%	1.28%	1.07%	1.48%	0.41%	0.24%	0.14%	0.33%	5.40
Purbeck	1.47%	1.56%	1.19%	1.93%	2.58%	1.36%	1.02%	1.70%	0.30%	0.19%	0.05%	0.34%	7.02
Reading	2.81%	2.81%	2.57%	3.05%	4.43%	2.24%	2.02%	2.45%	1.09%	0.57%	0.45%	0.69%	3.92
Redbridge	0.97%	0.95%	0.86%	1.04%	1.57%	0.79%	0.71%	0.88%	0.34%	0.16%	0.12%	0.19%	5.05
Redcar and Cleveland	1.83%	2.06%	1.77%	2.35%	3.14%	1.78%	1.52%	2.05%	0.45%	0.28%	0.16%	0.39%	6.49
Redditch	1.71%	1.87%	1.58%	2.15%	2.56%	1.44%	1.19%	1.69%	0.80%	0.42%	0.29%	0.56%	3.42
Reigate and Banstead	1.62%	1.62%	1.41%	1.84%	2.65%	1.33%	1.14%	1.53%	0.56%	0.29%	0.20%	0.39%	4.55
Ribble Valley	1.25%	1.38%	1.06%	1.70%	2.09%	1.13%	0.85%	1.42%	0.38%	0.25%	0.10%	0.40%	4.53
Richmond upon Thames	1.68%	1.75%	1.53%	1.97%	2.79%	1.47%	1.27%	1.67%	0.57%	0.28%	0.20%	0.37%	5.18
Richmondshire	1.38%	1.48%	1.12%	1.84%	2.15%	1.21%	0.88%	1.53%	0.53%	0.28%	0.12%	0.43%	4.38
Rochdale	2.57%	2.56%	2.34%	2.77%	4.02%	2.02%	1.83%	2.20%	1.04%	0.54%	0.44%	0.65%	3.73
Rochford	1.27%	1.28%	1.05%	1.51%	2.08%	1.07%	0.86%	1.28%	0.42%	0.22%	0.12%	0.31%	4.94
Rossendale	1.76%	1.85%	1.54%	2.16%	2.94%	1.60%	1.31%	1.89%	0.52%	0.25%	0.14%	0.35%	6.46
Rother	2.02%	2.17%	1.80%	2.54%	3.18%	1.73%	1.40%	2.06%	0.83%	0.44%	0.28%	0.61%	3.90
Rotherham	2.64%	2.83%	2.63%	3.04%	4.25%	2.36%	2.17%	2.55%	0.98%	0.48%	0.40%	0.55%	4.96
Rugby	1.72%	1.78%	1.53%	2.03%	2.64%	1.37%	1.15%	1.59%	0.77%	0.41%	0.29%	0.53%	3.35
Runnymede	2.75%	2.77%	2.36%	3.17%	4.40%	2.29%	1.92%	2.66%	1.00%	0.48%	0.31%	0.64%	4.77
Rushcliffe	1.44%	1.44%	1.23%	1.65%	2.07%	1.05%	0.87%	1.23%	0.77%	0.39%	0.28%	0.50%	2.71
Rushmoor	1.59%	1.75%	1.43%	2.06%	2.60%	1.44%	1.16%	1.72%	0.51%	0.30%	0.16%	0.45%	4.73
Rutland	1.31%	1.62%	1.12%	2.13%	2.15%	1.37%	0.91%	1.84%	0.45%	0.25%	0.07%	0.43%	5.53
Ryedale	0.87%	1.17%	0.82%	1.52%	1.49%	1.02%	0.70%	1.35%	0.24%	0.15%	0.03%	0.27%	6.90
Salford	1.45%	1.57%	1.38%	1.76%	2.30%	1.30%	1.12%	1.47%	0.55%	0.28%	0.20%	0.35%	4.69
Sandwell	0.71%	0.73%	0.64%	0.81%	1.21%	0.63%	0.55%	0.71%	0.19%	0.09%	0.06%	0.12%	6.90
Scarborough	1.18%	1.43%	1.15%	1.71%	1.89%	1.15%	0.90%	1.40%	0.42%	0.28%	0.15%	0.42%	4.08
Sedgemoor	1.00%	1.01%	0.81%	1.20%	1.69%	0.87%	0.69%	1.05%	0.29%	0.13%	0.07%	0.20%	6.51
Sefton	1.91%	1.98%	1.81%	2.15%	3.06%	1.62%	1.46%	1.77%	0.72%	0.36%	0.29%	0.43%	4.47
Selby	1.09%	1.18%	0.92%	1.44%	1.73%	0.96%	0.72%	1.20%	0.43%	0.22%	0.11%	0.33%	4.38
Sevenoaks	2.50%	2.65%	2.37%	2.94%	4.00%	2.18%	1.92%	2.44%	0.94%	0.47%	0.35%	0.59%	4.59
Sheffield	2.47%	2.43%	2.30%	2.56%	3.87%	1.96%	1.85%	2.08%	0.99%	0.47%	0.41%	0.52%	4.22
Shepway	3.23%	3.37%	3.05%	3.70%	4.90%	2.57%	2.29%	2.85%	1.51%	0.80%	0.64%	0.96%	3.20
Shropshire	1.36%	1.46%	1.30%	1.62%	2.17%	1.20%	1.06%	1.35%	0.51%	0.26%	0.20%	0.33%	4.58
Slough	1.45%	1.42%	1.26%	1.58%	2.35%	1.19%	1.04%	1.33%	0.52%	0.23%	0.17%	0.30%	5.07
Solihull	3.46%	3.38%	3.15%	3.61%	5.16%	2.59%	2.39%	2.79%	1.65%	0.79%	0.68%	0.91%	3.26
South Bucks	1.34%	1.40%	1.13%	1.68%	2.19%	1.16%	0.91%	1.41%	0.47%	0.24%	0.13%	0.36%	4.78
South Cambridgeshire	1.39%	1.69%	1.45%	1.92%	2.21%	1.35%	1.14%	1.56%	0.52%	0.34%	0.23%	0.45%	3.99
South Derbyshire	1.76%	1.81%	1.55%	2.08%	2.96%	1.56%	1.31%	1.81%	0.51%	0.25%	0.15%	0.35%	6.27
South Gloucestershire	1.42%	1.52%	1.37%	1.66%	2.35%	1.27%	1.14%	1.41%	0.47%	0.24%	0.18%	0.30%	5.24

©2021 Roman-Urrestarazu A et al. *JAMA Pediatrics*.

South Hams	1.31%	1.31%	1.07%	1.54%	2.22%	1.15%	0.93%	1.37%	0.36%	0.16%	0.08%	0.23%	7.28
South Holland	1.68%	1.71%	1.44%	1.97%	2.73%	1.44%	1.20%	1.69%	0.57%	0.27%	0.17%	0.37%	5.43
South Kesteven	1.71%	1.84%	1.63%	2.06%	2.91%	1.57%	1.37%	1.78%	0.53%	0.27%	0.19%	0.35%	5.82
South Lakeland	1.68%	1.73%	1.47%	2.00%	2.75%	1.43%	1.18%	1.67%	0.59%	0.31%	0.20%	0.42%	4.63
South Norfolk	1.00%	0.95%	0.79%	1.10%	1.57%	0.76%	0.62%	0.90%	0.39%	0.18%	0.11%	0.25%	4.15
South Northamptonshire	1.45%	1.41%	1.19%	1.63%	2.28%	1.13%	0.93%	1.32%	0.58%	0.28%	0.18%	0.38%	3.98
South Oxfordshire	1.81%	1.79%	1.56%	2.01%	3.07%	1.51%	1.31%	1.71%	0.53%	0.28%	0.19%	0.37%	5.44
South Ribble	1.51%	1.66%	1.38%	1.94%	2.45%	1.35%	1.10%	1.60%	0.53%	0.31%	0.18%	0.45%	4.34
South Somerset	1.01%	1.09%	0.91%	1.26%	1.66%	0.88%	0.73%	1.04%	0.35%	0.20%	0.12%	0.28%	4.32
South Staffordshire	1.86%	1.95%	1.67%	2.23%	3.00%	1.60%	1.35%	1.86%	0.68%	0.35%	0.23%	0.46%	4.62
South Tyneside	1.73%	1.69%	1.48%	1.90%	2.84%	1.41%	1.22%	1.61%	0.61%	0.28%	0.20%	0.35%	5.13
Southampton	1.87%	1.92%	1.70%	2.13%	2.94%	1.53%	1.34%	1.72%	0.75%	0.39%	0.29%	0.49%	3.95
Southend-on-Sea	1.35%	1.39%	1.23%	1.55%	2.28%	1.20%	1.05%	1.36%	0.36%	0.19%	0.12%	0.25%	6.49
Southwark	2.71%	2.75%	2.55%	2.94%	4.51%	2.30%	2.13%	2.48%	0.89%	0.44%	0.37%	0.52%	5.18
Spelthorne	2.17%	2.21%	1.89%	2.52%	3.42%	1.77%	1.49%	2.05%	0.84%	0.43%	0.29%	0.58%	4.11
St Albans	2.02%	2.03%	1.83%	2.24%	3.23%	1.68%	1.49%	1.86%	0.78%	0.36%	0.28%	0.44%	4.70
St Edmundsbury	1.49%	1.54%	1.30%	1.78%	2.49%	1.30%	1.08%	1.52%	0.47%	0.24%	0.15%	0.34%	5.33
St. Helens	2.02%	2.21%	1.98%	2.45%	3.24%	1.77%	1.57%	1.98%	0.76%	0.44%	0.33%	0.55%	4.02
Stafford	2.13%	2.32%	2.04%	2.59%	3.43%	1.92%	1.66%	2.17%	0.82%	0.40%	0.29%	0.51%	4.79
Staffordshire Moorlands	0.99%	1.05%	0.84%	1.26%	1.59%	0.89%	0.69%	1.08%	0.37%	0.16%	0.09%	0.24%	5.43
Stevenage	2.56%	2.44%	2.16%	2.73%	3.96%	1.96%	1.70%	2.22%	1.06%	0.48%	0.36%	0.61%	4.05
Stockport	1.65%	1.68%	1.51%	1.84%	2.61%	1.35%	1.20%	1.50%	0.62%	0.32%	0.25%	0.40%	4.18
Stockton-on-Tees	1.67%	1.86%	1.65%	2.08%	2.68%	1.52%	1.32%	1.72%	0.62%	0.34%	0.25%	0.44%	4.42
Stoke-on-Trent	0.94%	1.00%	0.86%	1.13%	1.52%	0.83%	0.71%	0.96%	0.35%	0.16%	0.11%	0.21%	5.13
Stratford-on-Avon	2.11%	2.13%	1.88%	2.39%	3.29%	1.67%	1.45%	1.90%	0.91%	0.46%	0.34%	0.58%	3.64
Stroud	1.09%	1.03%	0.86%	1.20%	1.89%	0.92%	0.76%	1.08%	0.25%	0.11%	0.06%	0.17%	8.01
Suffolk Coastal	1.79%	1.80%	1.57%	2.02%	2.73%	1.39%	1.19%	1.58%	0.85%	0.41%	0.31%	0.52%	3.36
Sunderland	2.74%	2.78%	2.58%	2.98%	4.33%	2.24%	2.06%	2.42%	1.09%	0.54%	0.45%	0.63%	4.12
Surrey Heath	1.45%	1.43%	1.18%	1.67%	2.39%	1.21%	0.98%	1.43%	0.46%	0.22%	0.12%	0.31%	5.51
Sutton	1.90%	1.89%	1.72%	2.05%	3.00%	1.53%	1.38%	1.68%	0.77%	0.35%	0.28%	0.42%	4.36
Swale	2.62%	2.70%	2.46%	2.95%	4.03%	2.16%	1.94%	2.38%	1.14%	0.54%	0.44%	0.65%	3.97
Swindon	2.07%	2.13%	1.93%	2.33%	3.29%	1.71%	1.53%	1.89%	0.78%	0.42%	0.33%	0.51%	4.08
Tameside	1.10%	1.18%	1.02%	1.35%	1.88%	1.03%	0.88%	1.19%	0.28%	0.15%	0.09%	0.21%	6.92
Tamworth	2.21%	2.33%	2.01%	2.65%	3.50%	1.86%	1.58%	2.15%	0.85%	0.47%	0.32%	0.62%	3.99
Tandridge	2.34%	2.49%	2.14%	2.84%	3.49%	1.90%	1.60%	2.21%	1.19%	0.58%	0.42%	0.75%	3.27
Taunton Deane	0.81%	1.02%	0.76%	1.28%	1.24%	0.79%	0.56%	1.02%	0.37%	0.23%	0.11%	0.35%	3.46
Teignbridge	1.55%	1.63%	1.41%	1.85%	2.43%	1.31%	1.12%	1.51%	0.62%	0.32%	0.22%	0.42%	4.13
Telford and Wrekin	1.76%	1.87%	1.66%	2.07%	2.92%	1.57%	1.38%	1.76%	0.58%	0.29%	0.21%	0.38%	5.34
Tendring	2.26%	2.24%	2.00%	2.49%	3.51%	1.79%	1.57%	2.01%	0.97%	0.45%	0.35%	0.56%	3.94
Test Valley	1.11%	1.25%	1.00%	1.50%	1.90%	1.05%	0.83%	1.27%	0.30%	0.20%	0.09%	0.32%	5.16
Tewkesbury	0.89%	0.91%	0.72%	1.10%	1.25%	0.67%	0.50%	0.84%	0.53%	0.24%	0.15%	0.33%	2.80
Thanet	2.51%	2.65%	2.40%	2.91%	4.02%	2.16%	1.93%	2.39%	0.91%	0.49%	0.38%	0.61%	4.37
Three Rivers	1.50%	1.45%	1.23%	1.67%	2.66%	1.34%	1.12%	1.55%	0.25%	0.12%	0.05%	0.18%	11.44
Thurrock	1.28%	1.39%	1.21%	1.57%	2.15%	1.20%	1.03%	1.37%	0.36%	0.19%	0.12%	0.26%	6.28
Tonbridge and Malling	1.84%	1.92%	1.70%	2.15%	3.08%	1.62%	1.42%	1.83%	0.59%	0.30%	0.21%	0.39%	5.37
Torbay	1.24%	1.25%	1.06%	1.44%	2.01%	1.04%	0.87%	1.22%	0.42%	0.21%	0.13%	0.29%	4.97
Torridge	1.17%	1.22%	0.92%	1.52%	1.72%	0.90%	0.64%	1.15%	0.58%	0.32%	0.16%	0.48%	2.80
Tower Hamlets	1.70%	1.62%	1.48%	1.75%	2.79%	1.32%	1.19%	1.44%	0.61%	0.30%	0.24%	0.36%	4.46
Trafford	1.03%	1.12%	0.98%	1.25%	1.62%	0.89%	0.77%	1.01%	0.42%	0.23%	0.17%	0.29%	3.93
Tunbridge Wells	1.84%	1.87%	1.64%	2.10%	3.15%	1.63%	1.41%	1.84%	0.49%	0.25%	0.16%	0.33%	6.64
Uttlesford	1.11%	1.09%	0.89%	1.29%	1.87%	0.95%	0.76%	1.14%	0.31%	0.13%	0.07%	0.20%	7.17
Vale of White Horse	2.07%	2.12%	1.86%	2.37%	3.18%	1.66%	1.43%	1.89%	0.92%	0.46%	0.34%	0.58%	3.62
Wakefield	1.81%	1.86%	1.71%	2.02%	2.96%	1.55%	1.40%	1.69%	0.62%	0.32%	0.25%	0.38%	4.86
Walsall	1.46%	1.52%	1.38%	1.65%	2.40%	1.26%	1.14%	1.38%	0.48%	0.25%	0.20%	0.31%	4.96
Waltham Forest	1.65%	1.72%	1.57%	1.88%	2.63%	1.39%	1.25%	1.52%	0.63%	0.34%	0.27%	0.41%	4.09
Wandsworth	2.38%	2.51%	2.30%	2.71%	3.80%	2.03%	1.84%	2.22%	0.91%	0.48%	0.38%	0.57%	4.26
Warrington	1.22%	1.30%	1.14%	1.46%	1.95%	1.04%	0.90%	1.18%	0.46%	0.26%	0.18%	0.34%	3.99
Warwick	2.00%	2.14%	1.89%	2.40%	3.11%	1.68%	1.46%	1.91%	0.84%	0.46%	0.34%	0.58%	3.68
Watford	1.43%	1.33%	1.14%	1.53%	2.21%	1.08%	0.90%	1.26%	0.60%	0.25%	0.17%	0.33%	4.27
Waveney	2.14%	2.23%	1.92%	2.53%	3.63%	1.90%	1.62%	2.18%	0.63%	0.33%	0.21%	0.44%	5.85
Waverley	1.87%	2.67%	2.26%	3.09%	3.02%	2.23%	1.86%	2.61%	0.65%	0.44%	0.26%	0.61%	5.10
Wealden	1.72%	1.70%	1.50%	1.91%	2.74%	1.39%	1.20%	1.58%	0.63%	0.31%	0.22%	0.41%	4.43
Wellingborough	1.53%	1.65%	1.37%	1.93%	2.30%	1.24%	1.00%	1.48%	0.75%	0.41%	0.27%	0.55%	2.99
Welwyn Hatfield	1.83%	1.86%	1.62%	2.10%	2.96%	1.51%	1.30%	1.73%	0.69%	0.35%	0.24%	0.45%	4.37
West Berkshire	3.17%	3.20%	2.94%	3.45%	5.28%	2.71%	2.48%	2.94%	0.95%	0.48%	0.38%	0.58%	5.63
West Devon	1.27%	1.36%	1.04%	1.68%	2.03%	1.12%	0.83%	1.41%	0.48%	0.24%	0.11%	0.37%	4.66
West Dorset	2.69%	2.78%	2.46%	3.10%	4.36%	2.30%	2.00%	2.60%	0.99%	0.48%	0.35%	0.61%	4.81
West Lancashire	1.95%	2.15%	1.84%	2.47%	3.06%	1.72%	1.45%	1.99%	0.74%	0.44%	0.29%	0.59%	3.94
West Lindsey	3.11%	3.30%	2.94%	3.67%	4.86%	2.65%	2.33%	2.98%	1.28%	0.65%	0.49%	0.81%	4.08
West Oxfordshire	1.48%	1.37%	1.17%	1.57%	2.38%	1.14%	0.95%	1.33%	0.55%	0.23%	0.15%	0.30%	5.02
West Somerset	0.91%	0.99%	0.57%	1.41%	1.56%	0.79%	0.44%	1.15%	0.25%	0.20%	0.00%	0.41%	4.03
Westminster	1.88%	1.83%	1.61%	2.06%	3.02%	1.48%	1.28%	1.68%	0.71%	0.35%	0.25%	0.45%	4.23
Weymouth and Portland	3.09%	3.08%	2.66%	3.50%	4.73%	2.38%	2.01%	2.75%	1.40%	0.70%	0.50%	0.90%	3.41
Wigan	1.02%	1.03%	0.91%	1.16%	1.67%	0.88%	0.76%	0.99%	0.33%	0.16%	0.11%	0.21%	5.53
Wiltshire	2.19%	2.10%	1.98%	2.22%	3.39%	1.65%	1.54%	1.76%	0.95%	0.45%	0.39%	0.50%	3.67
Winchester	1.02%	1.29%	1.00%	1.58%	1.71%	1.12%	0.85%	1.39%	0.28%	0.17%	0.07%	0.27%	6.58
Windsor and Maidenhead	1.88%	1.93%	1.71%	2.15%	2.96%	1.57%	1.37%	1.77%	0.74%	0.35%	0.26%	0.44%	4.44
Wirral	2.29%	2.36%	2.20%	2.52%	3.70%	1.93%	1.79%	2.07%	0.84%	0.43%	0.36%	0.50%	4.50
Woking	2.70%	2.72%	2.37%	3.06%	4.38%	2.24%	1.94%	2.55%	0.88%	0.48%	0.32%	0.63%	4.70
Wokingham	2.34%	2.35%	2.14%	2.56%	3.78%	1.93%	1.74%	2.11%	0.83%	0.42%	0.33%	0.51%	4.59
Wolverhampton	0.80%	0.90%	0.79%	1.02%	1.36%	0.79%	0.68%	0.89%	0.22%	0.12%	0.08%	0.16%	6.65

©2021 Roman-Urrestarazu A et al. *JAMA Pediatrics*.

Worcester	1.25%	1.48%	1.19%	1.76%	2.00%	1.19%	0.93%	1.44%	0.46%	0.29%	0.16%	0.42%	4.09
Worthing	1.79%	1.91%	1.59%	2.23%	2.82%	1.58%	1.29%	1.87%	0.66%	0.33%	0.20%	0.47%	4.73
Wychavon	1.36%	1.35%	1.14%	1.56%	2.20%	1.08%	0.90%	1.26%	0.49%	0.27%	0.17%	0.37%	3.96
Wycombe	1.41%	1.46%	1.30%	1.62%	2.28%	1.19%	1.05%	1.34%	0.48%	0.27%	0.19%	0.34%	4.41
Wyre	1.18%	1.21%	0.96%	1.45%	1.89%	0.95%	0.74%	1.16%	0.42%	0.25%	0.13%	0.38%	3.78
Wyre Forest	1.19%	1.20%	0.98%	1.42%	1.90%	0.99%	0.79%	1.19%	0.43%	0.21%	0.12%	0.31%	4.62
York	1.03%	1.14%	0.97%	1.30%	1.69%	0.95%	0.79%	1.10%	0.35%	0.19%	0.12%	0.26%	4.97

©2021 Roman-Urrestarazu A et al. *JAMA Pediatrics*.

Table 5. Age and Sex Specified Prevalence for SEND Statement and Support by Local Area District.

	ASD SEND Statement		ASD SEND Support	
LAD	Adjusted Prevalence	Confidence Interval	Adjusted Prevalence	Confidence Interval
Newham	0.40%	0.35 – 0.45%	0.96%	0.96 – 1.03%
Rushcliffe	0.46%	0.38 – 0.55%	0.89%	0.89 – 1.02%
Forest Heath	0.33%	0.21 – 0.46%	0.62%	0.62 – 0.79%
Nottingham	0.91%	0.83 – 0.98%	1.65%	1.65 – 1.76%
Mid Suffolk	0.39%	0.29 – 0.48%	0.69%	0.69 – 0.81%
Coventry	1.08%	1 .00– 1.15%	1.82%	1.82 – 1.92%
Babergh	0.63%	0.51 – 0.75%	1.05%	1.05 – 1.22%
Ashfield	1.03%	0.91 – 1.14%	1.67%	1.67 – 1.84%
Torridge	0.46%	0.32 – 0.59%	0.74%	0.74 – 0.91%
Stevenage	0.89%	0.75 – 1.02%	1.43%	1.43 – 1.60%
Mansfield	0.89%	0.76 – 1.02%	1.43%	1.43 – 1.60%
Gedling	0.85%	0.74 – 0.96%	1.36%	1.36 – 1.52%
North Hertfordshire	0.82%	0.71 – 0.93%	1.27%	1.27 – 1.41%
St Albans	0.77%	0.68 – 0.86%	1.19%	1.19 – 1.31%
Broxtowe	0.62%	0.51 – 0.72%	0.92%	0.92 – 1.06%
Dacorum	0.62%	0.53 – 0.71%	0.93%	0.93 – 1.04%
Newark and Sherwood	0.87%	0.76 – 0.98%	1.27%	1.27 – 1.43%
Bassetlaw	0.60%	0.50 – 0.70%	0.88%	0.88 – 1.01%
Rotherham	1.19%	1.10 – 1.28%	1.64%	1.64 – 1.75%
West Dorset	1.09%	0.94 – 1.23%	1.47%	1.47 – 1.66%
Lincoln	1.41%	1.25 – 1.58%	1.91%	1.91 – 2.11%
Suffolk Coastal	0.76%	0.65 – 0.87%	1.00%	1.00 – 1.14%
Stafford	0.99%	0.87 – 1.11%	1.30%	1.30 – 1.45%
Vale of White Horse	0.87%	0.74 – 0.99%	1.13%	1.13 – 1.26%
Liverpool	1.32%	1.25 – 1.39%	1.71%	1.71 – 1.80%
Hart	0.71%	0.57 – 0.86%	0.90%	0.90 – 1.06%
North Dorset	0.90%	0.74 – 1.06%	1.08%	1.08 – 1.27%
Ipswich	0.92%	0.82 – 1.03%	1.10%	1.10 – 1.22%
West Berkshire	1.43%	1.30 – 1.56%	1.68%	1.68 – 1.82%
Tamworth	1.03%	0.88 – 1.17%	1.20%	1.20 – 1.37%
East Hertfordshire	0.61%	0.53 – 0.69%	0.71%	0.71 – 0.81%
Hertsmere	0.67%	0.56 – 0.79%	0.74%	0.74 – 0.86%
Solihull	1.62%	1.50 – 1.73%	1.75%	1.75 – 1.88%
West Oxfordshire	0.61%	0.50 – 0.72%	0.66%	0.66 – 0.77%
Welwyn Hatfield	0.85%	0.73 – 0.97%	0.91%	0.91 – 1.04%
Bromsgrove	0.91%	0.78 – 1.05%	0.94%	0.94 – 1.08%
Lichfield	0.82%	0.70 – 0.94%	0.84%	0.84 – 0.98%
Redditch	0.87%	0.73 – 1.00%	0.88%	0.88 – 1.02%
Wiltshire	0.96%	0.89 – 1.02%	0.95%	0.95 – 1.01%
Mendip	0.78%	0.67 – 0.90%	0.77%	0.77 – 0.89%
Malvern Hills	0.78%	0.63 – 0.93%	0.77%	0.77 – 0.92%
South Oxfordshire	0.79%	0.68 – 0.91%	0.78%	0.78 – 0.89%
East Devon	0.64%	0.53 – 0.74%	0.62%	0.62 – 0.72%
Gravesham	1.64%	1.49 – 1.80%	1.58%	1.58 – 1.74%
West Lindsey	1.73%	1.55 – 1.91%	1.66%	1.66 – 1.84%
Sheffield	1.22%	1.15 – 1.29%	1.15%	1.15 – 1.22%
North Kesteven	0.92%	0.80 – 1.04%	0.86%	0.86 – 0.99%
Shepway	1.64%	1.48 – 1.81%	1.55%	1.55 – 1.72%
East Northamptonshire	0.79%	0.66 – 0.92%	0.74%	0.74 – 0.87%
St. Helens	1.24%	1.12 – 1.36%	1.16%	1.16 – 1.28%
Three Rivers	0.70%	0.58 – 0.82%	0.65%	0.65 – 0.77%
Harrogate	0.86%	0.76 – 0.96%	0.80%	0.80 – 0.91%
Cherwell	0.75%	0.66 – 0.85%	0.69%	0.69 – 0.79%
Watford	0.65%	0.54 – 0.76%	0.59%	0.59 – 0.70%
South Staffordshire	1.07%	0.93 – 1.20%	0.96%	0.96 – 1.11%

©2021 Roman-Urrestarazu A et al. *JAMA Pediatrics*.

Gosport	0.58%	0.46 – 0.71%	0.52%	0.52 – 0.64%
Craven	0.98%	0.80 – 1.17%	0.88%	0.88 – 1.06%
Sedgemoor	0.66%	0.55 – 0.78%	0.59%	0.59 – 0.70%
Corby	0.59%	0.46 – 0.71%	0.52%	0.52 – 0.64%
Plymouth	1.37%	1.27 – 1.47%	1.20%	1.20 – 1.30%
Cotswold	0.29%	0.20 – 0.38%	0.25%	0.25 – 0.34%
St Edmundsbury	0.74%	0.62 – 0.86%	0.64%	0.64 – 0.76%
Ashford	0.99%	0.88 – 1.11%	0.85%	0.85 – 0.96%
Tendring	1.19%	1.06 – 1.33%	1.01%	1.01 – 1.14%
Ribble Valley	0.75%	0.59 – 0.91%	0.63%	0.63 – 0.78%
Rushmoor	1.11%	0.92 – 1.30%	0.93%	0.93 – 1.10%
Richmondshire	0.98%	0.78 – 1.17%	0.82%	0.82 – 1.00%
Boston	0.64%	0.51 – 0.78%	0.54%	0.54 – 0.66%
Lancaster	1.24%	1.11 – 1.37%	1.03%	1.03 – 1.15%
Rossendale	1.13%	0.97 – 1.30%	0.93%	0.93 – 1.08%
Oxford	0.96%	0.83 – 1.08%	0.78%	0.78 – 0.89%
Wokingham	1.22%	1.10 – 1.33%	0.99%	0.99 – 1.09%
South Somerset	0.73%	0.64 – 0.83%	0.59%	0.59 – 0.68%
Surrey Heath	0.78%	0.65 – 0.92%	0.62%	0.62 – 0.74%
Fareham	0.36%	0.27 – 0.46%	0.29%	0.29 – 0.37%
Cannock Chase	1.36%	1.19 – 1.52%	1.06%	1.06 – 1.21%
Dartford	1.64%	1.49 – 1.80%	1.29%	1.29 – 1.44%
North West Leicestershire	0.83%	0.70 – 0.95%	0.65%	0.65 – 0.75%
Gateshead	0.90%	0.80 – 1.00%	0.71%	0.71 – 0.80%
Lewisham	1.72%	1.62 – 1.83%	1.35%	1.35 – 1.45%
North Somerset	0.55%	0.48 – 0.62%	0.43%	0.43 – 0.49%
Bristol, City of	0.98%	0.91 – 1.05%	0.76%	0.76 – 0.83%
Walsall	0.83%	0.76 – 0.90%	0.64%	0.64 – 0.71%
Bromley	1.13%	1.05 – 1.21%	0.87%	0.87 – 0.94%
Horsham	0.79%	0.68 – 0.91%	0.61%	0.61 – 0.71%
South Holland	1.03%	0.89 – 1.18%	0.79%	0.79 – 0.92%
Derbyshire Dales	0.90%	0.75 – 1.06%	0.69%	0.69 – 0.84%
Test Valley	0.88%	0.69 – 1.06%	0.66%	0.66 – 0.81%
Canterbury	1.18%	1.05 – 1.30%	0.89%	0.89 – 1.00%
Warwick	1.16%	1.03 – 1.29%	0.88%	0.88 – 1.00%
Central Bedfordshire	1.27%	1.18 – 1.36%	0.95%	0.95 – 1.03%
Wyre Forest	0.64%	0.52 – 0.75%	0.48%	0.48 – 0.58%
Waveney	1.39%	1.22 – 1.56%	1.02%	1.02 – 1.17%
Sevenoaks	1.53%	1.38 – 1.68%	1.12%	1.12 – 1.26%
Medway	1.74%	1.64 – 1.83%	1.27%	1.27 – 1.36%
South Tyneside	1.03%	0.91 – 1.15%	0.75%	0.75 – 0.85%
Stockton-on-Tees	1.26%	1.13 – 1.38%	0.91%	0.91 – 1.01%
Reading	1.53%	1.40 – 1.67%	1.10%	1.10 – 1.22%
South Norfolk	0.77%	0.65 – 0.90%	0.55%	0.55 – 0.67%
Bolsover	0.99%	0.82 – 1.15%	0.71%	0.71 – 0.85%
King's Lynn and West Norfolk	1.18%	1.05 – 1.32%	0.85%	0.85 – 0.96%
Hartlepool	1.14%	0.96 – 1.31%	0.81%	0.81 – 0.96%
Bexley	1.31%	1.22 – 1.41%	0.94%	0.94 – 1.02%
Weymouth and Portland	1.97%	1.73 – 2.20%	1.38%	1.38 – 1.61%
Dover	1.04%	0.91 – 1.18%	0.73%	0.73 – 0.84%
South Kesteven	1.06%	0.95 – 1.18%	0.74%	0.74 – 0.84%
South Derbyshire	1.02%	0.88 – 1.16%	0.71%	0.71 – 0.83%
Sunderland	1.74%	1.62 – 1.85%	1.21%	1.21 – 1.31%
Runnymede	1.70%	1.46 – 1.93%	1.18%	1.18 – 1.37%
South Hams	0.71%	0.58 – 0.84%	0.49%	0.49 – 0.61%
Dudley	0.73%	0.66 – 0.81%	0.51%	0.51 – 0.57%
Amber Valley	1.34%	1.20 – 1.49%	0.92%	0.92 – 1.04%
Wirral	1.37%	1.29 – 1.46%	0.94%	0.94 – 1.02%
Isle of Wight	1.27%	1.14 – 1.40%	0.86%	0.86 – 0.98%
North East Derbyshire	0.85%	0.72 – 0.98%	0.58%	0.58 – 0.70%
Bracknell Forest	1.51%	1.36 – 1.66%	1.03%	1.03 – 1.16%

©2021 Roman-Urrestarazu A et al. *JAMA Pediatrics*.

Hillingdon	1.31%	1.22 – 1.40%	0.89%	0.89 – 0.97%
New Forest	0.83%	0.72 – 0.94%	0.56%	0.56 – 0.65%
Eastbourne	1.57%	1.34 – 1.80%	1.07%	1.07 – 1.26%
Tunbridge Wells	1.02%	0.89 – 1.15%	0.68%	0.68 – 0.79%
West Devon	0.65%	0.49 – 0.81%	0.44%	0.44 – 0.58%
Breckland	0.86%	0.74 – 0.98%	0.57%	0.57 – 0.67%
Cornwall	0.81%	0.76 – 0.87%	0.54%	0.54 – 0.59%
Rochdale	1.81%	1.66 – 1.96%	1.19%	1.19 – 1.30%
West Somerset	0.62%	0.40 – 0.84%	0.41%	0.41 – 0.60%
Greenwich	1.38%	1.28 – 1.47%	0.90%	0.90 – 0.98%
Kingston upon Hull, City of	1.28%	1.15 – 1.41%	0.83%	0.83 – 0.94%
Sefton	1.23%	1.14 – 1.32%	0.79%	0.79 – 0.87%
Kingston upon Thames	1.14%	1.03 – 1.24%	0.73%	0.73 – 0.81%
Blackburn with Darwen	0.92%	0.81 – 1.03%	0.58%	0.58 – 0.67%
Wellingborough	0.92%	0.78 – 1.06%	0.57%	0.57 – 0.69%
Worcester	1.06%	0.90 – 1.22%	0.66%	0.66 – 0.79%
Reigate and Banstead	1.04%	0.91 – 1.16%	0.64%	0.64 – 0.74%
Stratford-on-Avon	1.29%	1.15 – 1.44%	0.79%	0.79 – 0.91%
Harborough	0.71%	0.60 – 0.82%	0.43%	0.43 – 0.54%
Newcastle upon Tyne	0.93%	0.85 – 1.01%	0.57%	0.57 – 0.64%
Swale	1.67%	1.53 – 1.80%	1.01%	1.01 – 1.12%
Windsor and Maidenhead	1.14%	1.01 – 1.26%	0.69%	0.69 – 0.79%
Christchurch	0.67%	0.50 – 0.85%	0.41%	0.41 – 0.54%
Daventry	0.94%	0.80 – 1.08%	0.56%	0.56 – 0.69%
Wychavon	0.75%	0.63 – 0.86%	0.45%	0.45 – 0.55%
Bedford	1.14%	1.03 – 1.25%	0.68%	0.68 – 0.77%
County Durham	1.08%	1.01 – 1.14%	0.64%	0.64 – 0.69%
Wakefield	1.24%	1.15 – 1.33%	0.74%	0.74 – 0.81%
Bath and North East Somerset	1.19%	1.07 – 1.30%	0.70%	0.70 – 0.80%
Peterborough	1.09%	0.99 – 1.18%	0.64%	0.64 – 0.72%
East Staffordshire	1.02%	0.90 – 1.13%	0.60%	0.60 – 0.70%
Elmbridge	1.20%	1.05 – 1.35%	0.71%	0.71 – 0.83%
Darlington	1.30%	1.11 – 1.49%	0.77%	0.77 – 0.90%
Norwich	0.84%	0.72 – 0.95%	0.49%	0.49 – 0.59%
Southampton	1.26%	1.12 – 1.41%	0.74%	0.74 – 0.85%
Colchester	1.48%	1.34 – 1.62%	0.87%	0.87 – 0.97%
North Devon	1.01%	0.84 – 1.19%	0.59%	0.59 – 0.72%
North Warwickshire	1.26%	1.06 – 1.46%	0.73%	0.73 – 0.89%
Guildford	1.26%	1.11 – 1.40%	0.73%	0.73 – 0.84%
Poole	0.83%	0.73 – 0.93%	0.48%	0.48 – 0.55%
Bradford	0.85%	0.80 – 0.89%	0.48%	0.48 – 0.52%
Brentwood	1.36%	1.17 – 1.55%	0.78%	0.78 – 0.93%
East Dorset	0.68%	0.56 – 0.80%	0.39%	0.39 – 0.49%
Eastleigh	1.09%	0.87 – 1.31%	0.62%	0.62 – 0.75%
East Lindsey	1.57%	1.42 – 1.72%	0.88%	0.88 – 1.01%
Teignbridge	0.98%	0.86 – 1.10%	0.55%	0.55 – 0.65%
Tonbridge and Malling	1.26%	1.13 – 1.39%	0.70%	0.70 – 0.80%
South Gloucestershire	0.94%	0.86 – 1.02%	0.53%	0.53 – 0.59%
Charnwood	1.08%	0.96 – 1.19%	0.60%	0.60 – 0.69%
Basildon	1.30%	1.19 – 1.41%	0.72%	0.72 – 0.81%
Doncaster	1.16%	1.07 – 1.24%	0.64%	0.64 – 0.70%
Tameside	1.07%	0.95 – 1.20%	0.59%	0.59 – 0.68%
Great Yarmouth	2.64%	2.31 – 2.96%	1.44%	1.44 – 1.65%
Northampton	1.07%	0.98 – 1.16%	0.58%	0.58 – 0.65%
Mole Valley	1.06%	0.90 – 1.22%	0.58%	0.58 – 0.71%
Birmingham	1.68%	1.63 – 1.73%	0.91%	0.91 – 0.95%
Purbeck	1.07%	0.86 – 1.28%	0.58%	0.58 – 0.74%
Broxbourne	0.84%	0.71 – 0.96%	0.44%	0.44 – 0.54%
Brighton and Hove	1.05%	0.95 – 1.16%	0.55%	0.55 – 0.63%
Northumberland	1.34%	1.25 – 1.43%	0.69%	0.69 – 0.76%
Rochford	0.87%	0.73 – 1.01%	0.45%	0.45 – 0.55%

©2021 Roman-Urrestarazu A et al. *JAMA Pediatrics*.

Hambleton	0.74%	0.60 – 0.87%	0.38%	0.38 – 0.48%
Hastings	1.17%	0.99 – 1.35%	0.60%	0.60 – 0.73%
Erewash	1.10%	0.95 – 1.24%	0.56%	0.56 – 0.67%
Melton	1.11%	0.92 – 1.31%	0.56%	0.56 – 0.71%
Cheshire East	0.75%	0.69 – 0.81%	0.38%	0.38 – 0.42%
Adur	1.06%	0.89 – 1.24%	0.53%	0.53 – 0.66%
Wealden	1.19%	1.07 – 1.32%	0.59%	0.59 – 0.69%
Stroud	0.66%	0.55 – 0.76%	0.33%	0.33 – 0.41%
Luton	1.22%	1.11 – 1.32%	0.60%	0.60 – 0.68%
Leeds	0.70%	0.66 – 0.74%	0.35%	0.35 – 0.38%
Maldon	1.39%	1.18 – 1.59%	0.68%	0.68 – 0.83%
Spelthorne	1.45%	1.27 – 1.63%	0.71%	0.71 – 0.84%
York	0.83%	0.73 – 0.92%	0.41%	0.41 – 0.47%
Chesterfield	0.82%	0.69 – 0.95%	0.40%	0.40 – 0.49%
Crawley	0.87%	0.76 – 0.99%	0.43%	0.43 – 0.51%
Maidstone	1.30%	1.19 – 1.42%	0.63%	0.63 – 0.72%
South Cambridgeshire	1.46%	1.32 – 1.61%	0.71%	0.71 – 0.81%
Burnley	0.97%	0.82 – 1.13%	0.47%	0.47 – 0.58%
North Norfolk	1.20%	1.02 – 1.38%	0.58%	0.58 – 0.71%
Derby	1.54%	1.43 – 1.64%	0.74%	0.74 – 0.81%
South Northamptonshire	0.60%	0.50 – 0.70%	0.28%	0.28 – 0.36%
Arun	0.93%	0.82 – 1.05%	0.44%	0.44 – 0.52%
Calderdale	0.73%	0.65 – 0.80%	0.34%	0.34 – 0.40%
Woking	2.03%	1.82 – 2.24%	0.95%	0.95 – 1.10%
Telford and Wrekin	1.06%	0.95 – 1.17%	0.49%	0.49 – 0.57%
Barking and Dagenham	1.11%	1.02 – 1.19%	0.52%	0.52 – 0.58%
Cambridge	1.22%	1.03 – 1.41%	0.57%	0.57 – 0.70%
Tandridge	1.56%	1.36 – 1.76%	0.72%	0.72 – 0.86%
Croydon	1.41%	1.33 – 1.49%	0.64%	0.64 – 0.70%
Rugby	1.17%	1.03 – 1.31%	0.53%	0.53 – 0.64%
Sutton	1.25%	1.15 – 1.36%	0.57%	0.57 – 0.64%
Rutland	1.15%	0.91 – 1.40%	0.52%	0.52 – 0.71%
Torbay	0.85%	0.74 – 0.96%	0.38%	0.38 – 0.46%
Aylesbury Vale	1.18%	1.08 – 1.28%	0.53%	0.53 – 0.60%
Knowsley	1.35%	1.22 – 1.49%	0.60%	0.60 – 0.69%
Milton Keynes	1.11%	1.03 – 1.19%	0.49%	0.49 – 0.54%
Lewes	1.20%	1.02 – 1.38%	0.53%	0.53 – 0.64%
Bournemouth	1.20%	1.08 – 1.32%	0.52%	0.52 – 0.61%
Mid Sussex	1.19%	1.06 – 1.32%	0.52%	0.52 – 0.60%
Chiltern	0.83%	0.71 – 0.96%	0.35%	0.35 – 0.43%
Uttlesford	0.84%	0.71 – 0.97%	0.36%	0.36 – 0.45%
Copeland	1.51%	1.29 – 1.73%	0.64%	0.64 – 0.77%
Mid Devon	0.92%	0.75 – 1.09%	0.38%	0.38 – 0.49%
Herefordshire, County of	1.31%	1.17 – 1.46%	0.54%	0.54 – 0.64%
Cheshire West and Chester	1.29%	1.20 – 1.37%	0.52%	0.52 – 0.58%
Hinckley and Bosworth	1.17%	1.04 – 1.29%	0.47%	0.47 – 0.57%
Braintree	1.60%	1.46 – 1.75%	0.65%	0.65 – 0.75%
Richmond upon Thames	1.22%	1.10 – 1.35%	0.49%	0.49 – 0.57%
Kettering	1.10%	0.97 – 1.23%	0.44%	0.44 – 0.53%
Stoke-on-Trent	0.95%	0.85 – 1.04%	0.37%	0.37 – 0.43%
Scarborough	1.25%	1.08 – 1.42%	0.50%	0.50 – 0.60%
North Lincolnshire	0.91%	0.78 – 1.04%	0.35%	0.35 – 0.43%
Nuneaton and Bedworth	1.94%	1.76 – 2.11%	0.74%	0.74 – 0.85%
Swindon	1.81%	1.67 – 1.94%	0.68%	0.68 – 0.76%
Epsom and Ewell	1.29%	1.14 – 1.45%	0.48%	0.48 – 0.59%
Preston	1.44%	1.24 – 1.63%	0.53%	0.53 – 0.63%
Oldham	2.23%	2.08 – 2.37%	0.82%	0.82 – 0.91%
Wolverhampton	0.67%	0.61 – 0.74%	0.25%	0.25 – 0.29%
Southwark	2.06%	1.94 – 2.18%	0.75%	0.75 – 0.82%
High Peak	1.56%	1.37 – 1.74%	0.56%	0.56 – 0.68%
Exeter	1.59%	1.35 – 1.83%	0.57%	0.57 – 0.71%

Huntingdonshire	1.28%	1.16 – 1.39%	0.46%	0.46 – 0.53%
Wycombe	1.06%	0.96 – 1.16%	0.38%	0.38 – 0.44%
Fenland	1.55%	1.38 – 1.73%	0.55%	0.55 – 0.66%
Blaby	1.22%	1.07 – 1.37%	0.43%	0.43 – 0.53%
Pendle	1.53%	1.32 – 1.74%	0.54%	0.54 – 0.65%
Newcastle-under-Lyme	1.10%	0.94 – 1.25%	0.38%	0.38 – 0.48%
Harrow	1.17%	1.08 – 1.26%	0.41%	0.41 – 0.46%
Barnsley	1.67%	1.53 – 1.82%	0.58%	0.58 – 0.66%
Manchester	1.54%	1.46 – 1.63%	0.53%	0.53 – 0.58%
Basingstoke and Deane	1.83%	1.62 – 2.04%	0.63%	0.63 – 0.73%
Allerdale	1.22%	1.06 – 1.38%	0.42%	0.42 – 0.51%
Haringey	1.36%	1.26 – 1.47%	0.47%	0.47 – 0.53%
Merton	1.27%	1.16 – 1.38%	0.43%	0.43 – 0.50%
Oadby and Wigston	1.08%	0.90 – 1.26%	0.36%	0.36 – 0.49%
Hyndburn	1.17%	1.00 – 1.33%	0.39%	0.39 – 0.49%
Broadland	0.86%	0.74 – 0.98%	0.29%	0.29 – 0.36%
Rother	1.76%	1.53 – 1.99%	0.59%	0.59 – 0.72%
Wandsworth	1.87%	1.75 – 2.00%	0.62%	0.62 – 0.69%
Ealing	1.16%	1.08 – 1.24%	0.38%	0.38 – 0.43%
Staffordshire Moorlands	0.85%	0.72 – 0.98%	0.28%	0.28 – 0.36%
North Tyneside	1.05%	0.95 – 1.15%	0.34%	0.34 – 0.40%
Thanet	2.16%	2.01 – 2.32%	0.71%	0.71 – 0.81%
Southend-on-Sea	1.11%	1.01 – 1.22%	0.36%	0.36 – 0.42%
East Riding of Yorkshire	0.82%	0.75 – 0.89%	0.26%	0.26 – 0.31%
Epping Forest	0.85%	0.73 – 0.97%	0.27%	0.27 – 0.33%
Halton	1.75%	1.60 – 1.90%	0.55%	0.55 – 0.64%
Chichester	0.90%	0.76 – 1.04%	0.27%	0.27 – 0.35%
Fylde	1.16%	0.95 – 1.37%	0.34%	0.34 – 0.45%
South Ribble	2.01%	1.74 – 2.27%	0.58%	0.58 – 0.70%
Wyre	1.51%	1.29 – 1.74%	0.44%	0.44 – 0.55%
Harlow	0.74%	0.61 – 0.87%	0.21%	0.21 – 0.28%
Islington	1.90%	1.76 – 2.05%	0.55%	0.55 – 0.63%
Carlisle	0.75%	0.63 – 0.86%	0.21%	0.21 – 0.28%
Wigan	1.13%	1.03 – 1.22%	0.32%	0.32 – 0.37%
Hackney	1.41%	1.30 – 1.52%	0.39%	0.39 – 0.44%
Chelmsford	1.20%	1.08 – 1.31%	0.33%	0.33 – 0.39%
South Lakeland	1.18%	1.02 – 1.35%	0.32%	0.32 – 0.41%
Kirklees	0.58%	0.53 – 0.63%	0.16%	0.16 – 0.18%
Enfield	1.08%	1.01 – 1.15%	0.29%	0.29 – 0.33%
Sandwell	0.61%	0.56 – 0.67%	0.16%	0.16 – 0.19%
Lambeth	1.71%	1.60 – 1.82%	0.45%	0.45 – 0.51%
Middlesbrough	1.58%	1.44 – 1.72%	0.42%	0.42 – 0.49%
Taunton Deane	2.29%	1.90 – 2.68%	0.60%	0.60 – 0.77%
Hounslow	1.12%	1.03 – 1.21%	0.29%	0.29 – 0.33%
Eden	0.75%	0.59 – 0.91%	0.19%	0.19 – 0.28%
Salford	1.73%	1.58 – 1.88%	0.44%	0.44 – 0.51%
Shropshire	1.42%	1.31 – 1.53%	0.36%	0.36 – 0.42%
Slough	1.09%	0.98 – 1.19%	0.28%	0.28 – 0.33%
Worthing	2.29%	1.99 – 2.59%	0.58%	0.58 – 0.72%
Castle Point	1.02%	0.87 – 1.16%	0.26%	0.26 – 0.34%
Forest of Dean	0.54%	0.42 – 0.66%	0.13%	0.13 – 0.20%
Ryedale	1.05%	0.87 – 1.23%	0.26%	0.26 – 0.37%
Redcar and Cleveland	2.36%	2.10 – 2.62%	0.58%	0.58 – 0.70%
Bury	1.18%	1.04 – 1.32%	0.29%	0.29 – 0.35%
Winchester	1.79%	1.46 – 2.11%	0.43%	0.43 – 0.57%
Stockport	1.56%	1.44 – 1.68%	0.37%	0.37 – 0.43%
Warrington	1.16%	1.06 – 1.26%	0.27%	0.27 – 0.32%
City of London	1.40%	0.02 – 2.78%	0.33%	0.33 – 0.96%
Waltham Forest	1.54%	1.45 – 1.64%	0.36%	0.36 – 0.41%
Havant	1.57%	1.34 – 1.80%	0.36%	0.36 – 0.46%
South Bucks	1.25%	1.07 – 1.44%	0.29%	0.29 – 0.38%

©2021 Roman-Urrestarazu A et al. *JAMA Pediatrics*.

West Lancashire	2.60%	2.36 – 2.84%	0.59%	0.59 – 0.70%
North East Lincolnshire	1.41%	1.25 – 1.58%	0.31%	0.31 – 0.38%
Isles of Scilly	2.71%	0.00 – 6.21%	0.60%	0.60 – 1.42%
Selby	1.15%	0.97 – 1.33%	0.25%	0.25 – 0.34%
Havering	0.98%	0.89 – 1.06%	0.21%	0.21 – 0.26%
East Cambridgeshire	2.15%	1.88 – 2.43%	0.46%	0.46 – 0.58%
Leicester	1.38%	1.29 – 1.47%	0.29%	0.29 – 0.33%
Barnet	1.31%	1.23 – 1.39%	0.27%	0.27 – 0.31%
Cheltenham	0.67%	0.56 – 0.78%	0.14%	0.14 – 0.19%
Kensington and Chelsea	1.57%	1.35 – 1.78%	0.32%	0.32 – 0.42%
Hammersmith and Fulham	1.57%	1.41 – 1.74%	0.31%	0.31 – 0.38%
Trafford	1.04%	0.95 – 1.12%	0.20%	0.20 – 0.24%
Blackpool	1.84%	1.62 – 2.07%	0.35%	0.35 – 0.44%
Bolton	1.41%	1.31 – 1.51%	0.27%	0.27 – 0.31%
Tewkesbury	0.77%	0.64 – 0.90%	0.14%	0.14 – 0.20%
Chorley	3.50%	3.07 – 3.93%	0.63%	0.63 – 0.77%
East Hampshire	2.27%	1.91 – 2.63%	0.40%	0.40 – 0.54%
Waverley	3.73%	3.40 – 4.07%	0.65%	0.65 – 0.79%
Westminster	1.58%	1.41 – 1.74%	0.25%	0.25 – 0.32%
Brent	1.26%	1.18 – 1.35%	0.19%	0.19 – 0.23%
Redbridge	0.84%	0.77 – 0.91%	0.13%	0.13 – 0.15%
Tower Hamlets	1.43%	1.33 – 1.54%	0.21%	0.21 – 0.25%
Portsmouth	1.58%	1.37 – 1.78%	0.23%	0.23 – 0.28%
Thurrock	1.44%	1.32 – 1.57%	0.20%	0.20 – 0.25%
Gloucester	0.75%	0.65 – 0.85%	0.10%	0.10 – 0.14%
Camden	1.50%	1.36 – 1.64%	0.18%	0.18 – 0.23%
Barrow-in-Furness	2.84%	2.48 – 3.20%	0.28%	0.28 – 0.38%

©2021 Roman-Urrestarazu A et al. *JAMA Pediatrics*.

Table 6. ASD Prevalence by LAD and MFR

LAD	Data[a]
**10 highest ASD prevalence[b]**	
Solihull	3.38 (3.15-3.61) [3.26:1]
Shepway	3.37 (3.05-3.70) [3.20:1]
Gravesham	3.36 (3.05-3.67) [3.88:1]
West Lindsey	3.30 (2.94-3.67) [4.08:1]
Great Yarmouth	3.25 (2.84-3.67) [5.14:1]
West Berkshire	3.20 (2.94-3.45) [5.63:1]
Lewisham	3.19 (2.99-3.38) [4.45:1]
Lincoln	3.14 (2.79-3.50) [3.39:1]
Weymouth and Portland	3.08 (2.66-3.50) [3.41:1]
Coventry	3.08 (2.90-3.25) [4.05:1]
**10 lowest ASD prevalence**	
Cotswold	0.63 (0.46-0.81)
Kirklees	0.65 (0.57-0.73)
Forest of Dean	0.69 (0.50-0.88)
Sandwell	0.73 (0.64-0.81)
Fareham	0.78 (0.60-0.97)
Eden	0.85 (0.59-1.11)
Carlisle	0.86 (0.68-1.04)
Cheltenham	0.87 (0.70-1.05)
Gloucester	0.90 (0.75-1.04)
Wolverhampton	0.90 (0.79-1.02)
**10 highest MFR**	
Burnley	12.87:1
Three Rivers	10.83:1
Fareham	10.32:1
East Riding	8.11:1
Stroud	7.59:1
Eastleigh	7.47:1
Chesterfield	7.42:1
North Som	7.26:1
Chorley	7.20:1
Corby	7.17:1
**10 highest EHCP prevalence**	
Waverley	3.73 (3.40-4.07)
Chorley	3.50 (3.07-3.93)
Barrow-in-Furness	2.84 (2.48-3.20)
Isles of Scilly	2.71 (0.00-6.21)
Great Yarmouth	2.64 (2.31-2.96)
West Lancashire	2.60 (2.36-2.84)
Redcar and Cleveland	2.36 (2.10-2.62)
Worthing	2.29 (1.90-2.68)
Taunton Deane	2.29 (1.99-2.59)
East Hampshire	2.27 (1.91-2.63)
**10 highest SSR**	
Barrow-in-Furness	10.15
Camden	8.30
Gloucester	7.48
Thurrock	7.23
Portsmouth	6.97
Tower Hamlets	6.88
Redbridge	6.70

©2021 Roman-Urrestarazu A et al. *JAMA Pediatrics*.

Brent	6.50
Westminster	6.19
Waverley	5.72
**10 highest support prevalence**	
Lincoln	1.91 (1.91-2.11)
Coventry	1.82 (1.82-1.92)
Solihull	1.75 (1.75-1.88)
Liverpool	1.71 (1.71-1.80)
West Berkshire	1.68 (1.68-1.82)
Ashfield	1.67 (1.67-1.84)
West Lindsey	1.66 (1.66-1.84)
Nottingham	1.65 (1.65-1.76)
Rotherham	1.64 (1.64-1.75)
Gravesham	1.58 (1.58-1.74)
**10 lowest EHCP prevalence**	
Cotswold	0.29 (0.20-0.38)
Forest Heath	0.33 (0.21-0.46)
Fareham	0.36 (0.27-0.46)
Mid Suffolk	0.39 (0.29-0.48)
Newham	0.40 (0.35-0.45)
Torridge	0.46 (0.38-0.55)
Rushcliffe	0.46 (0.32-0.59)
Forest of Dean	0.54 (0.42-0.66)
North Somerset	0.55 (0.48-0.62)
Kirklees	0.58 (0.46-0.71)
**10 lowest support prevalence**	
Gloucester	0.10 (0.10-0.14)
Redbridge	0.13 (0.13-0.20)
Forest of Dean	0.13 (0.13-0.15)
Cheltenham	0.14 (0.14-0.19)
Tewkesbury	0.14 (0.14-0.20)
Kirklees	0.16 (0.16-0.18)
Sandwell	0.16 (0.16-0.19)
Camden	0.18 (0.18-0.23)
Eden	0.19 (0.19-0.28)
Brent	0.19 (0.19-0.23)
**10 lowest SSR**	
Newham	0.42
Rushcliffe	0.52
Forest Heath	0.54
Nottingham	0.55
Mid Suffolk	0.56
Coventry	0.59
Babergh	0.60
Ashfield	0.61
Torridge	0.62
Stevenage	0.62

Abbreviations: EHCP, Education Health and Care Plans; LAD, Local Authority District; MFR, male-to-female ratio; SEND, Special Educational Needs and Disability; SSR, standardized statement–to-support ratio.

[a]Unless otherwise indicated, data are expressed as prevalence (95% CI).

[b]MFRs are given in brackets.

Figure 1. SPR spread of autism by sex compared learning difficulties by LAD.

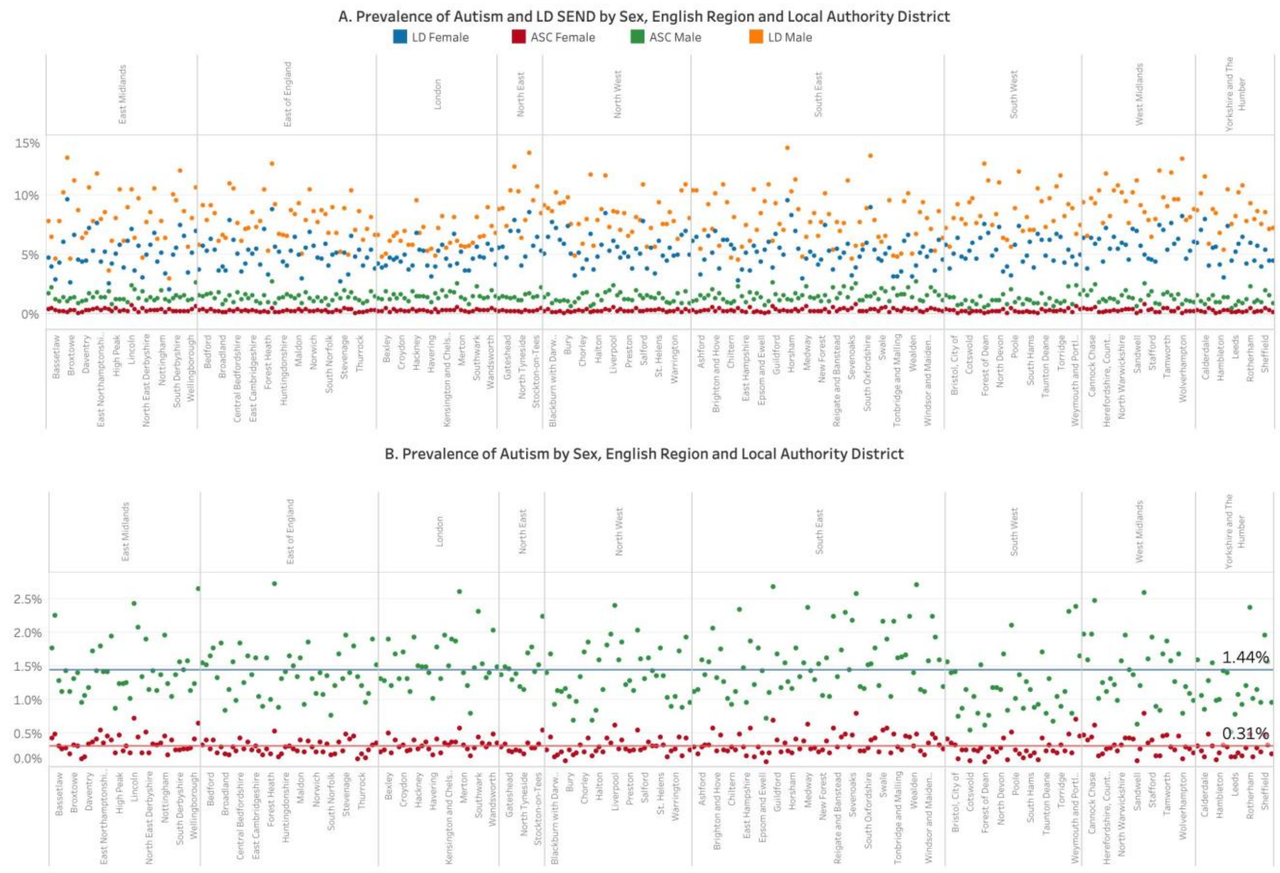

Figure 1A. Autism and Learning Difficult Prevalence by Region and Local Authority District (LAD) shows the prevalence spread across different English LAD, much more pronounced in learning difficulties. **Figure 1B.** Shows the prevalence variability found by sex across different LAD with less pronounced variability than learning difficulties, but visibly much more pronounced in the case of males.

©2021 Roman-Urrestarazu A et al. *JAMA Pediatrics*.

Figure 2. Variability of all other SEND categories by LAD.

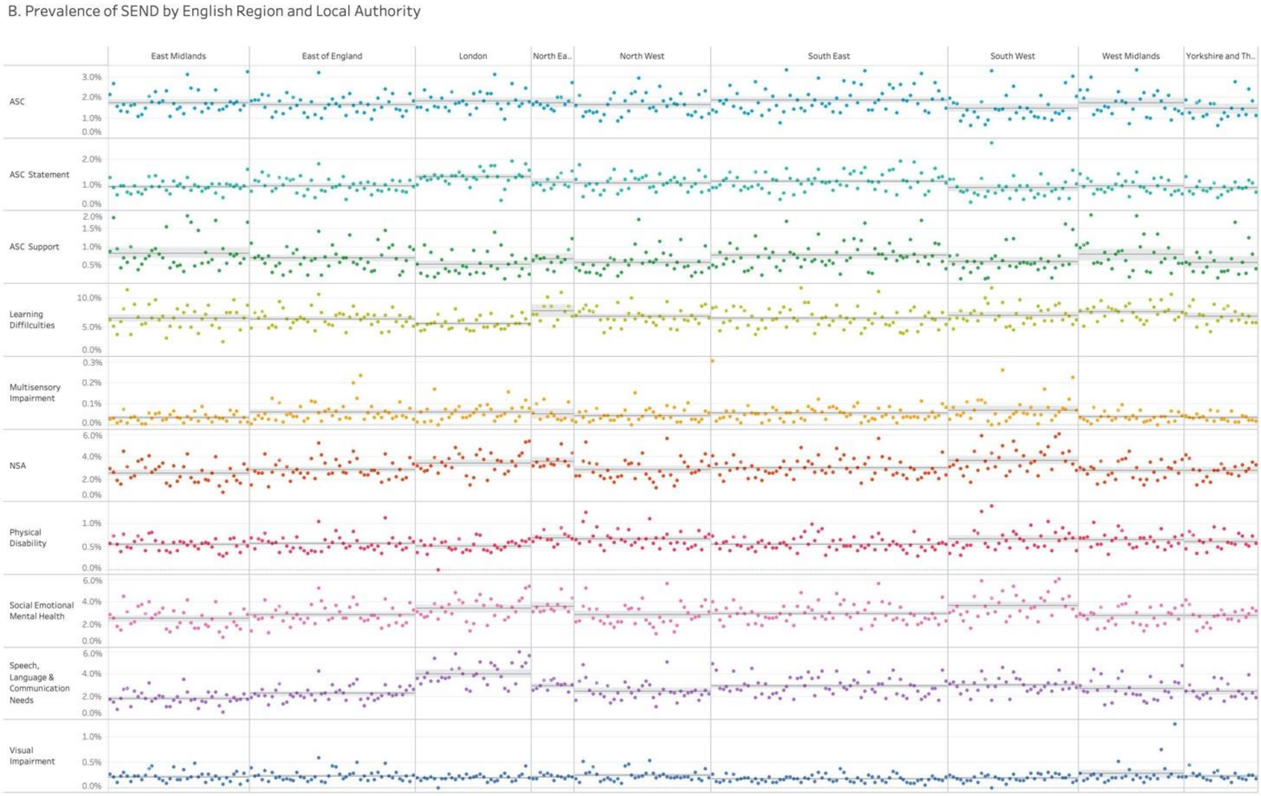

Figure 2. Shows in a scatter plot the variability of prevalence across 10 different SEND categories and how some SEND (such as visual impairment) are more stable compared to others others (such as Social, Emotional, and Mental Health).

©2021 Roman-Urrestarazu A et al. *JAMA Pediatrics*.

Figure 3. FSM Prevalence in England and London

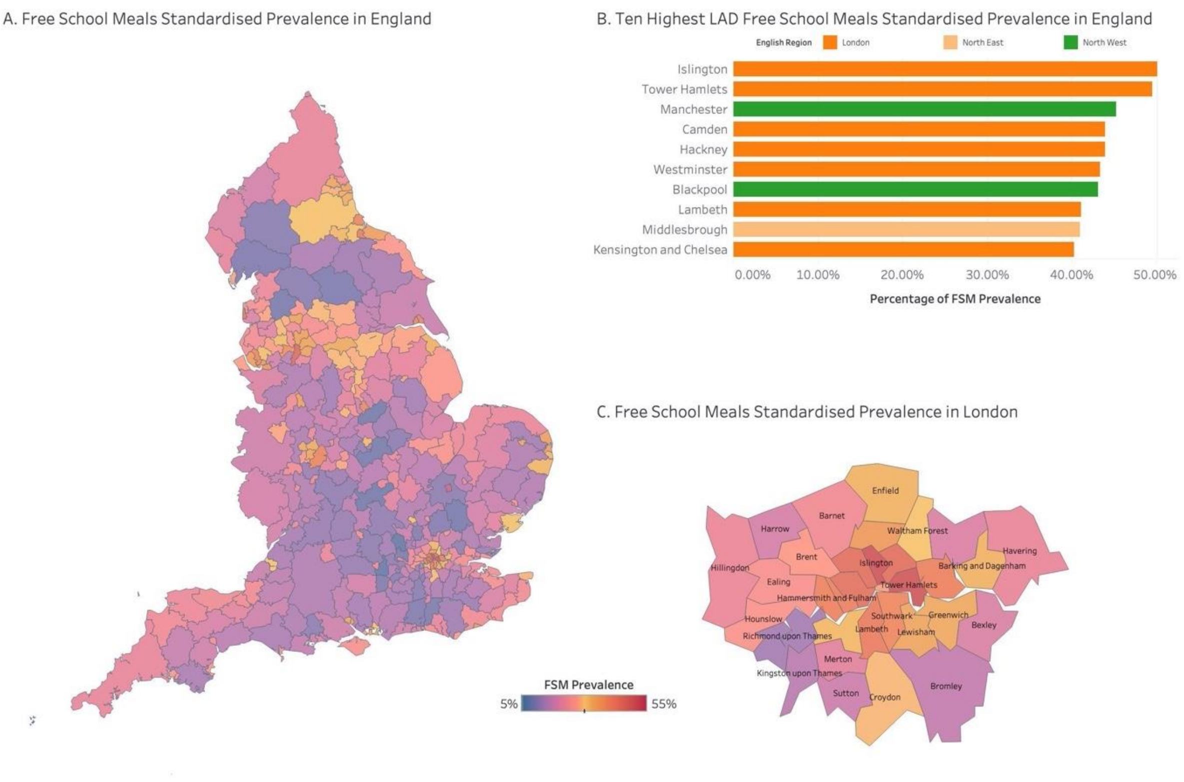

Figure 3A. FSM standardised prevalence in England by LAD. **Figure 3B.** Ten Highest FSM standardised prevalence by LAD and region **Figure 3C.** FSM standardised prevalence in London by LAD

©2021 Roman-Urrestarazu A et al. *JAMA Pediatrics*.

Figure 4. Standardised Prevalence of Autism by LAD, Male-to-Female-Ratio and Sex also reporting by support and EHCP

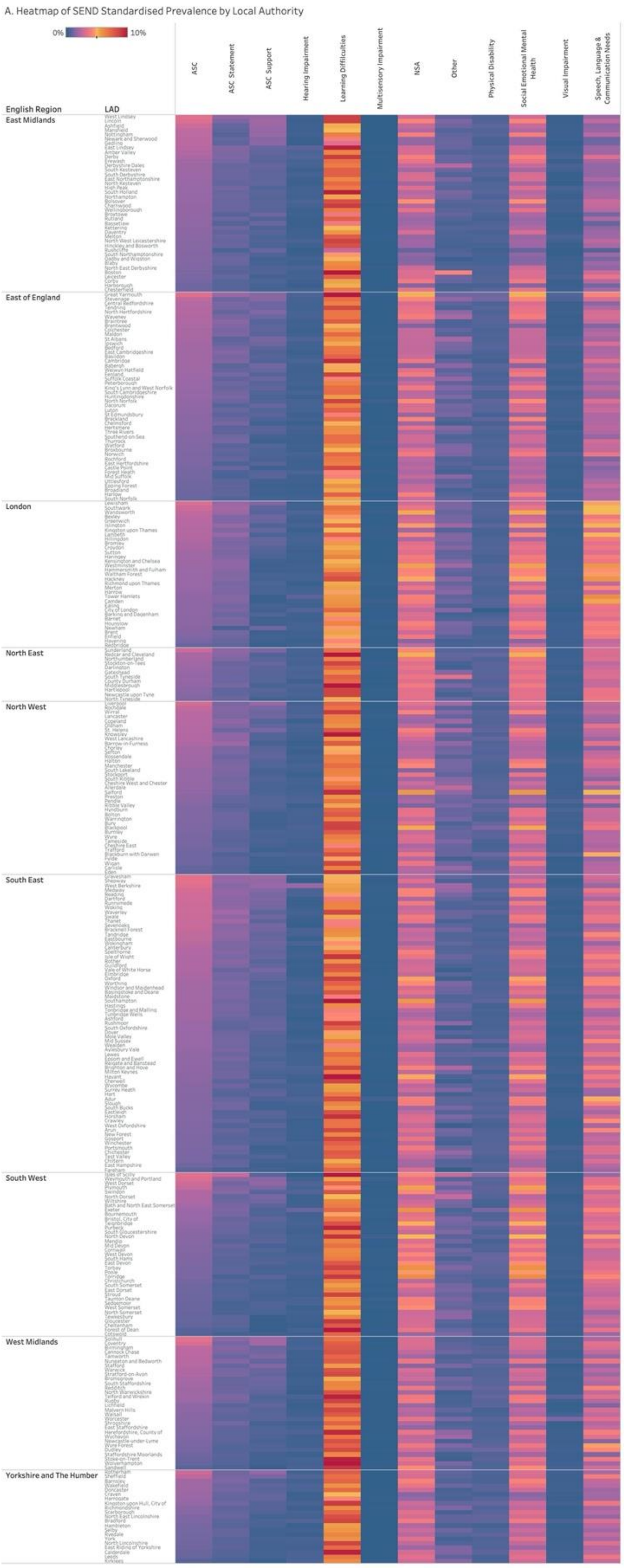

Figure 4. Shows a heatmap of all SEND standardized prevalence by Local Authority across all English regions where the predominance of learning disabilities over other SEND is clear, but where Social, Emotional, and Mental Health SEND also play a crucial role, even though it involves a more diffuse diagnostic pathway.

©2021 Roman-Urrestarazu A et al. *JAMA Pediatrics*.

Figure 5. Ratio of SEND statements versus SEN support in England.

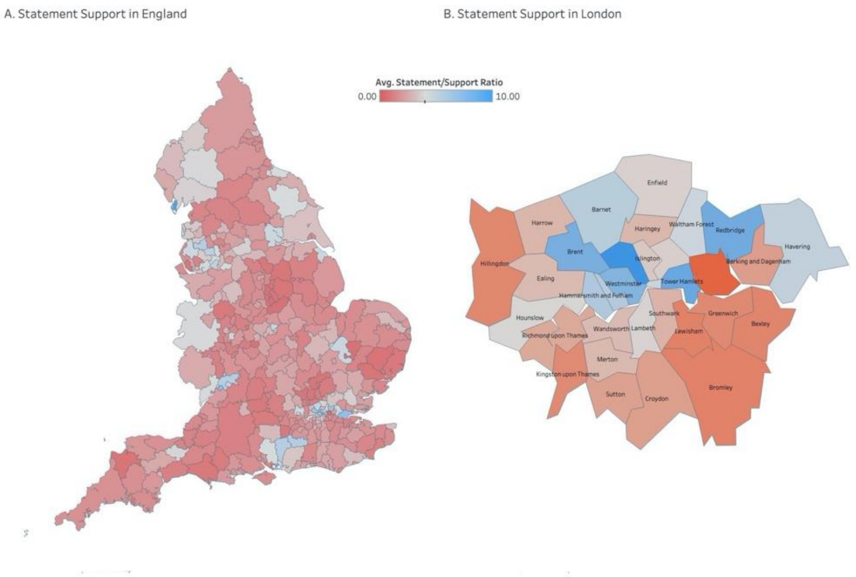

Figure 5A. Shows the ratio of SEND statements versus SEN support in England. **Figure 5B.** Specifies this ratio in London, where it appears that the ratio is more in favour of SEND support the Northern districts of London, while it is leans more towards SEND statements in the Southern districts.

Figure 6. Hypothesized model of the mediation analysis.

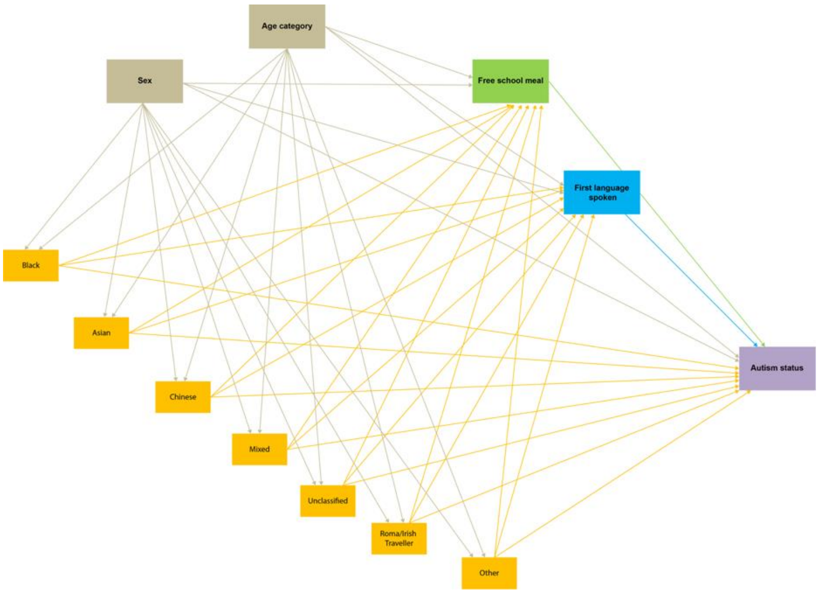

Figure 6. An illustration of the hypothesized model of the mediation analysis with sex and age as covariates and White pupils as the comparator group.

©2021 Roman-Urrestarazu A et al. *JAMA Pediatrics*.

Figure 7. Final model of the mediation analysis.

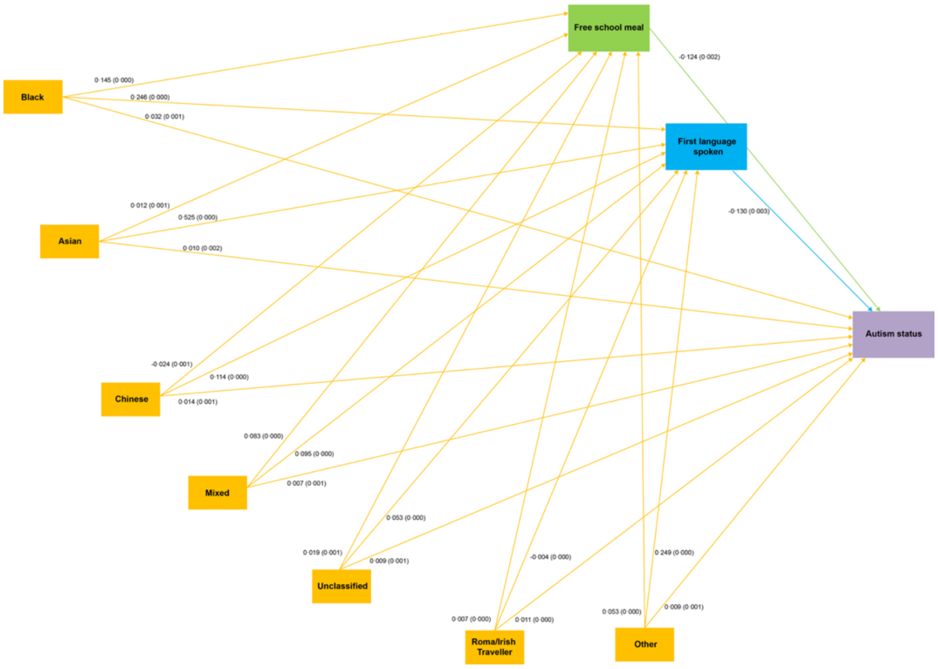

Figure 7. The final model of the mediation analysis presenting the direct effects of the observed characteristics on autism status.

©2021 Roman-Urrestarazu A et al. *JAMA Pediatrics*.

Figure 8. Indirect effects results

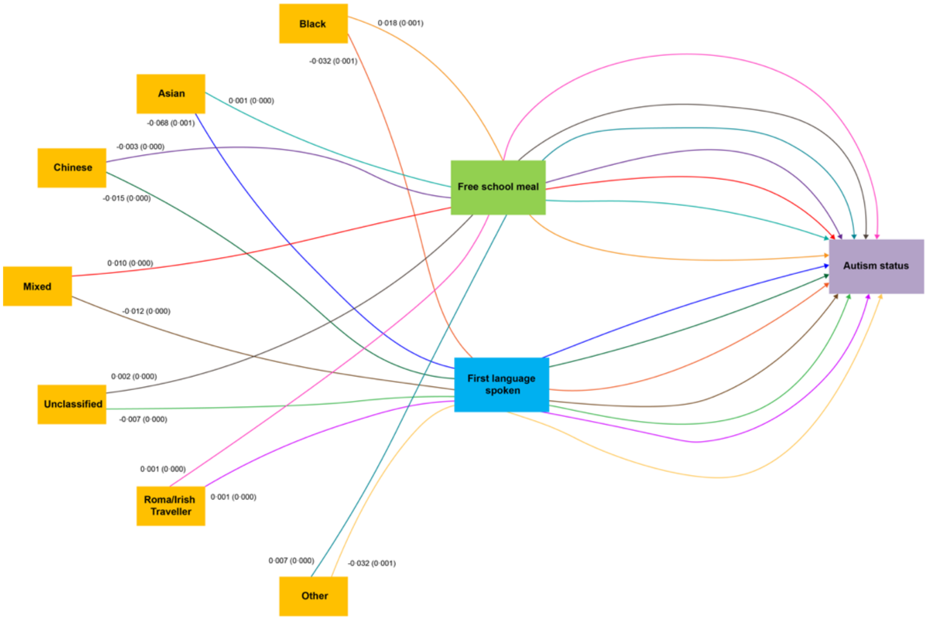

Figure 8. Indirect effects among ethnicity mediated through FSM and first language on autism status.

©2021 Roman-Urrestarazu A et al. *JAMA Pediatrics*.
